# Comparison of the Hepatotoxic Potential of Two Treatments for Autosomal-Dominant Polycystic Kidney DiseaseUsing Quantitative Systems Toxicology Modeling

**DOI:** 10.1007/s11095-019-2726-0

**Published:** 2020-01-06

**Authors:** J. L. Woodhead, L. Pellegrini, L. K. M. Shoda, B. A. Howell

**Affiliations:** 1 DILIsym Services, Inc., a Simulations Plus Company, Research Triangle Park, North Carolina USA; 2 Palladio Biosciences, Inc., Newtown, Pennsylvania USA

**Keywords:** Liver injury, quantitative systems toxicology, autosomal-dominant polycystic kidney disease, bile acids, mitochondria

## Abstract

**Purpose:**

Autosomal-dominant polycystic kidney disease (ADPKD) is an orphan disease with few current treatment options. The vasopressin V_2_ receptor antagonist tolvaptan is approved in multiple countries for the treatment of ADPKD, however its use is associated with clinically significant drug-induced liver injury.

**Methods:**

In prior studies, the potential for hepatotoxicity of tolvaptan was correctly predicted using DILIsym®, a quantitative systems toxicology (QST) mathematical model of drug-induced liver injury. In the current study, we evaluated lixivaptan, another proposed ADPKD treatment and vasopressin V_2_ receptor antagonist, using DILIsym®. Simulations were conducted that assessed the potential for lixivaptan and its three main metabolites to cause hepatotoxicity due to three injury mechanisms: bile acid accumulation, mitochondrial dysfunction, and oxidative stress generation. Results of these simulations were compared to previously published DILIsym results for tolvaptan.

**Results:**

No ALT elevations were predicted to occur at the proposed clinical dose for lixivaptan, in contrast to previously published simulation results for tolvaptan. As such, lixivaptan was predicted to have a markedly lower risk of hepatotoxicity compared to tolvaptan with respect to the hepatotoxicity mechanisms represented in DILIsym.

**Conclusions:**

These results demonstrate the potential for using QST methods to differentiate drugs in the same class for their potential to cause hepatotoxicity.

**Electronic supplementary material:**

The online version of this article (10.1007/s11095-019-2726-0) contains supplementary material, which is available to authorized users.

## Introduction

Lixivaptan is a selective vasopressin V_2_ receptor antagonist that was initially developed for the treatment of hyponatremia (1) and is currently being repurposed for the treatment of autosomal-dominant polycystic kidney disease (ADPKD). A similar drug within the same class, tolvaptan, caused liver enzyme elevations in two pivotal Phase III clinical trials in patients with ADPKD (2,3). Acute liver failure requiring liver transplantation has been reported in the post-marketing experience with tolvaptan, and frequent liver chemistry monitoring is recommended for patients taking tolvaptan for ADPKD (4). Despite these liver safety concerns, the unmet need for ADPKD treatment has supported tolvaptan’s approval in multiple countries, including the US. Considering the liver safety issues faced by tolvaptan, it is therefore desirable to compare the potential for liver toxicity due to lixivaptan to that observed for tolvaptan in order to determine whether lixivaptan could become a safer treatment option for the treatment of ADPKD.

Quantitative systems toxicology (QST) modeling combines knowledge about the effects of a drug in *in vitro* systems with knowledge about physiology to describe a drug’s potential for toxicity. Because of its ability to contextualize *in vitro* assay data, QST is an ideal candidate for comparing two drugs’ potential liabilities due to a particular set of mechanisms. DILIsym®, a QST platform model of drug-induced liver injury, has been successful in describing the differences between three hepatotoxic/non-hepatotoxic compound pairs: tolcapone and entacapone (5), bosentan and telmisartan (6), and troglitazone and pioglitazone (7). DILIsym uses QST modeling to assess liver toxicity risk due to bile acid transporter inhibition, mitochondrial dysfunction, and oxidative stress (8), i.e. three mechanisms that together account for the majority of all known cases of DILI. DILIsym was previously used to investigate the mechanisms behind tolvaptan-induced liver injury (9), determining that bile acid transporter inhibition and inhibition of the mitochondrial electron transport chain contribute to the observed liver enzyme elevations. A potential role for DM-4103, a long-lived metabolite of tolvaptan, was also identified (7).

In this paper, a DILIsym representation was constructed for lixivaptan and its three major metabolites, WAY-138451, WAY-141624, and WAY-138758, and was used to predict the potential risk of hepatotoxicity and likely margin of safety for lixivaptan at doses intended for the treatment of ADPKD. The simulation results for lixivaptan were compared to the previously published results for tolvaptan with a particular focus on the predictions made about the relative safety of the two drugs based on the mechanisms of toxicity included in DILIsym.

## Methods

DILIsym version 6A was used for all simulations described in this paper. The Lixivaptan-Specific SimPops and the Lixivaptan-Sensitive SimCohorts have been provided as supplemental materials; they will allow users with DILIsym v6A to reproduce the results in this paper.

### PBPK Modeling of Lixivaptan

#### General Approach and Use of Data

Lixivaptan and its three main metabolites were represented with the Physiologically-Based Pharmacokinetic (PBPK) sub-model within DILIsym with the goal of reasonably approximating liver exposure upon oral administration. The DILIsym PBPK sub-model framework used for lixivaptan consists of a compartmental model of the body with compartments for blood, gut, liver, muscle, and other tissues and has been described in depth elsewhere (6,7,10). The design and optimization process for the lixivaptan PBPK model is described in Supplement A.

#### Generation of SimPops

A customized SimPops including clinically relevant variability in lixivaptan exposure was created for this project. Parameter values related strictly to pharmacokinetics that were expected to vary among individuals were determined and ranges were assigned to these parameters. Details regarding the construction of the lixivaptan-specific SimPops, including the parameters varied and the distributions used for these parameters, are given in Supplement A.

16 individuals out of the 285-individual custom SimPops were selected as part of a Lixivaptan-Sensitive SimCohorts™. These simulated individuals were identified by escalating the lixivaptan dose to a supratherapeutic level in order to observe signs of simulated toxicity, and then selecting the 8 individuals with the highest ALT values. These individuals were added to the four individuals with the highest liver bile acid concentrations and the four individuals with the lowest electron transport chain activity in the simulations. These 16 simulated individuals were used for the dose escalation simulations.

In addition, bile acid transporter sensitivity analysis simulations were performed on the v4A_1_Multi16 SimCohorts. This SimCohorts is included in DILIsym version 6A and is comprised of 13 individuals who are the most susceptible to the various hepatotoxicity mechanisms in DILIsym, combined with 2 less-susceptible individuals and the baseline individual. As this SimCohorts contains the individuals most likely to respond to most drugs, it is generally used as a screening tool for sensitivity analyses; if no response is observed in this SimCohorts, it is unlikely that a response will appear in the full SimPops. In the case of this work, exposure variability in lixivaptan was not added to the v4A_1_Multi16 SimCohorts.

In the tolvaptan study, two custom SimPops were constructed, one that included clinically relevant variability in tolvaptan exposure (similar to the Lixivaptan-specific SimPops) and one for individuals with renal impairment due to the wider range of exposure variability observed in those individuals (9). However, data in patients with End Stage Renal Disease administered lixivaptan suggested that, unlike tolvaptan, lixivaptan exposure (AUC) and peak concentration (C_max_) were each decreased by approximately 30% in renal failure patients compared to subjects with normal renal function (personal communication; proprietary data not shown). In light of this, it was determined that the customized Lixivaptan-specific SimPops created based on normal healthy volunteer studies adequately captured the expected exposure variability in patients with renal impairment, and therefore would suffice for the prediction of toxicity in both normal healthy volunteers and ADPKD populations.

### *In Vitro* Assessment of Hepatotoxicity Mechanisms

Lixivaptan simulations required collecting data from *in vitro* systems to describe the relationships between lixivaptan and its metabolites with various mechanisms of hepatotoxicity. For each of these molecules, bile acid transporter inhibition, mitochondrial toxicity, and oxidative stress generation were assessed. The results from these assays were translated into toxicity parameters that were used as inputs into DILIsym. The details of the *in vitro* assays and their results are described in detail in Supplement B; the translation of these results into toxicity parameters is discussed in the Results section.

### Hepatotoxicity Simulations Conducted

#### Primary Clinical Protocols Simulated and Clinical Endpoints for Comparison of Lixivaptan and Tolvaptan Mediated Hepatotoxicity

The primary lixivaptan clinical protocols simulated throughout this analysis were as follows:

- 100 mg BID for 60 days (maximum dose intended for hyponatremia reported in historical FDA submission documents) (11,12);
- 200/100 mg split daily dosing for 12 weeks (proposed maximum chronic, clinical dose for ADPKD based on a pharmacodynamic endpoint assessment);
- 400 mg BID for 7 days (supratherapeutic dose evaluated in Phase I and QT prolongation studies)

The primary criterion for comparing lixivaptan simulation results to clinical results and to previously reported simulation results for tolvaptan (9) was the frequency of clinically meaningful ALT elevations, i.e. elevations greater than 3 times the upper limit of normal (ULN). Within DILIsym, the ULN is 40 U/L for ALT. An imbalance in ALT elevations compared to placebo was not observed in previously conducted clinical trials with lixivaptan (13). With tolvaptan, ALT elevations greater than 3X ULN occurred in 7.8% of simulated patients (9) and in 4–6% of treated ADPKD patients (2,3); the simulated frequency was used as the comparator for lixivaptan.

#### Mechanistic Investigation Simulations of Lixivaptan

The mode of bile acid transporter inhibition has been shown to be important for the prediction of bile acid-mediated DILI; noncompetitive inhibition is more potent than competitive inhibition, with mixed inhibition lying on a spectrum between these poles (6,14). For this project, IC_50_ values were measured for the inhibition of bile acid transporters by lixivaptan and its metabolites; as such, mode of inhibition was not determined experimentally. Mixed inhibition with α = 5 was used as an estimate of the mode of bile acid transporter inhibition. In order to investigate the importance of mode of bile acid transporter inhibition to DILIsym predictions of lixivaptan, the proposed clinical dosing regimen was also simulated in the full 285-individual lixivaptan-specific SimPops with efflux bile acid transporter inhibition treated as noncompetitive and uptake bile acid transporter inhibition treated as competitive. These conditions represent the worst-case scenario; that is, the modes of inhibition most likely to cause bile acid accumulation and thus toxicity. Other simulations were conducted in the v4A_1_Multi16 SimCohorts where efflux transporter mode of inhibition was varied between competitive, noncompetitive, and mixed inhibition with α = 5. Further simulations were also conducted without lixivaptan-mediated ROS generation in order to determine the contribution of this mechanism to predicted ALT elevations from lixivaptan treatment.

#### Dose Escalation Simulations for Lixivaptan

In order to understand the potential margin of safety for lixivaptan treatment, dose escalation simulations were conducted using the 16-individual Lixivaptan-Sensitive SimCohorts only. This approach was adopted to magnify a potential safety finding. These simulations were performed in the presence and absence of lixivaptan-generated and WAY-138451-generated ROS.

## Results

### PBPK Optimization Results

**Fig. 1:**
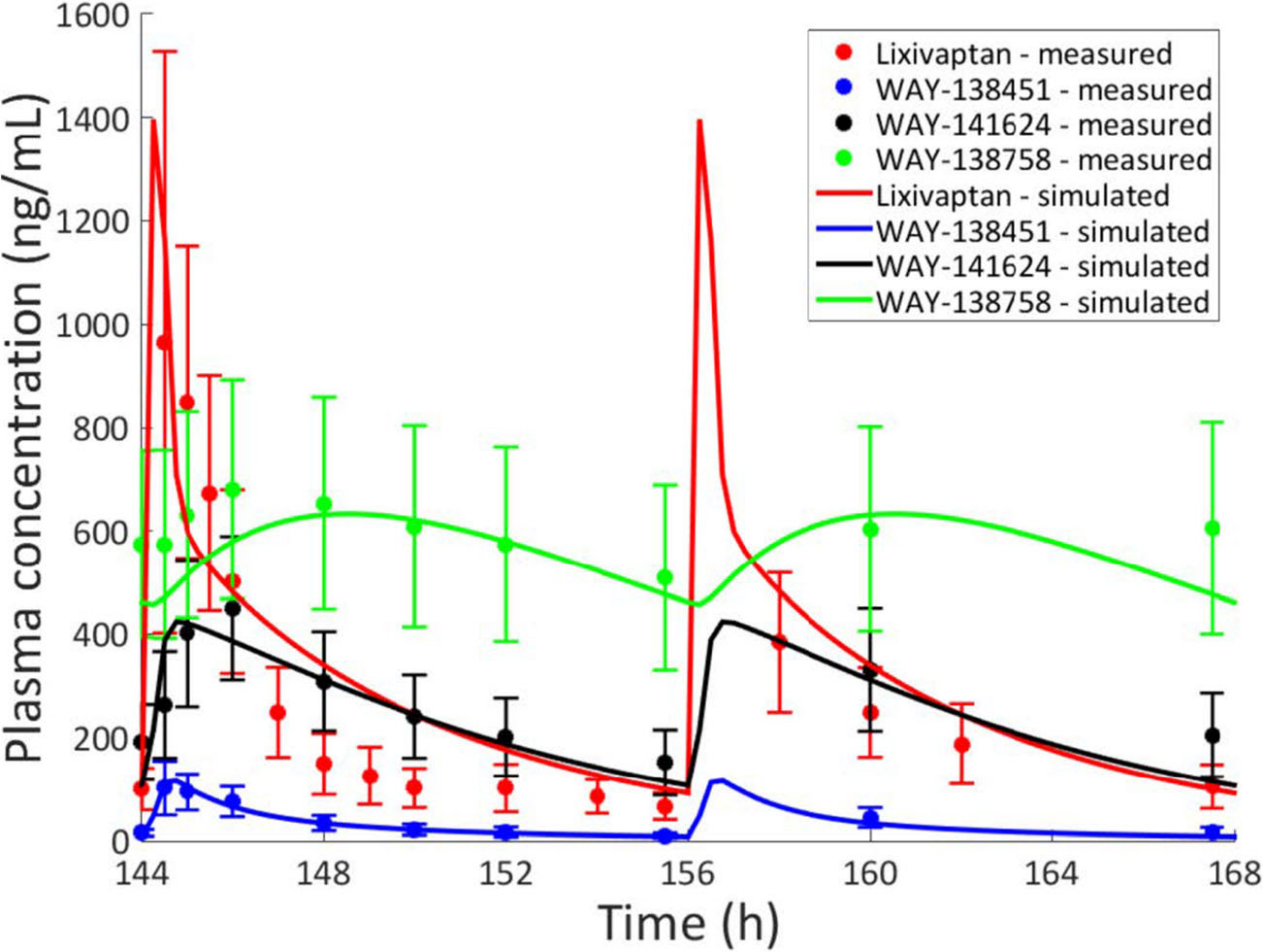
Simulated and measured (clinical study CK-LX1403) plasma concentrations of lixivaptan and its metabolites after 7 days of 100 mg BID dosing. Clinically, lixivaptan reached its steady-state concentration after 6 days (proprietary data not shown); the simulation is thus shown at that point.

**Fig. 2:**
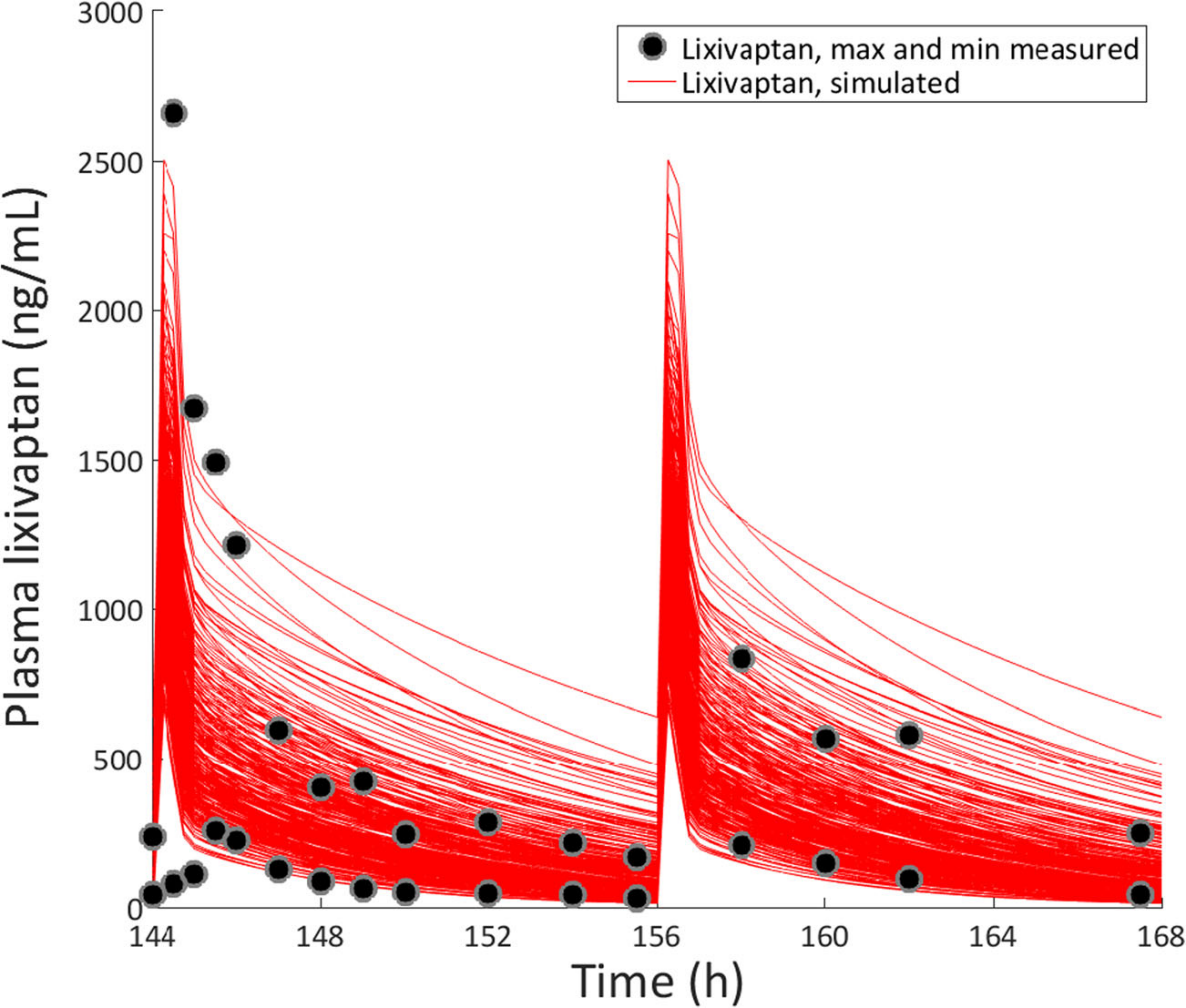
Simulated plasma time courses of lixivaptan from the customized SimPops on day 7 of 100 mg BID dosing compared with the maximum and minimum concentrations measured at each time point in clinical study CK-LX1403.

**Fig. 3:**
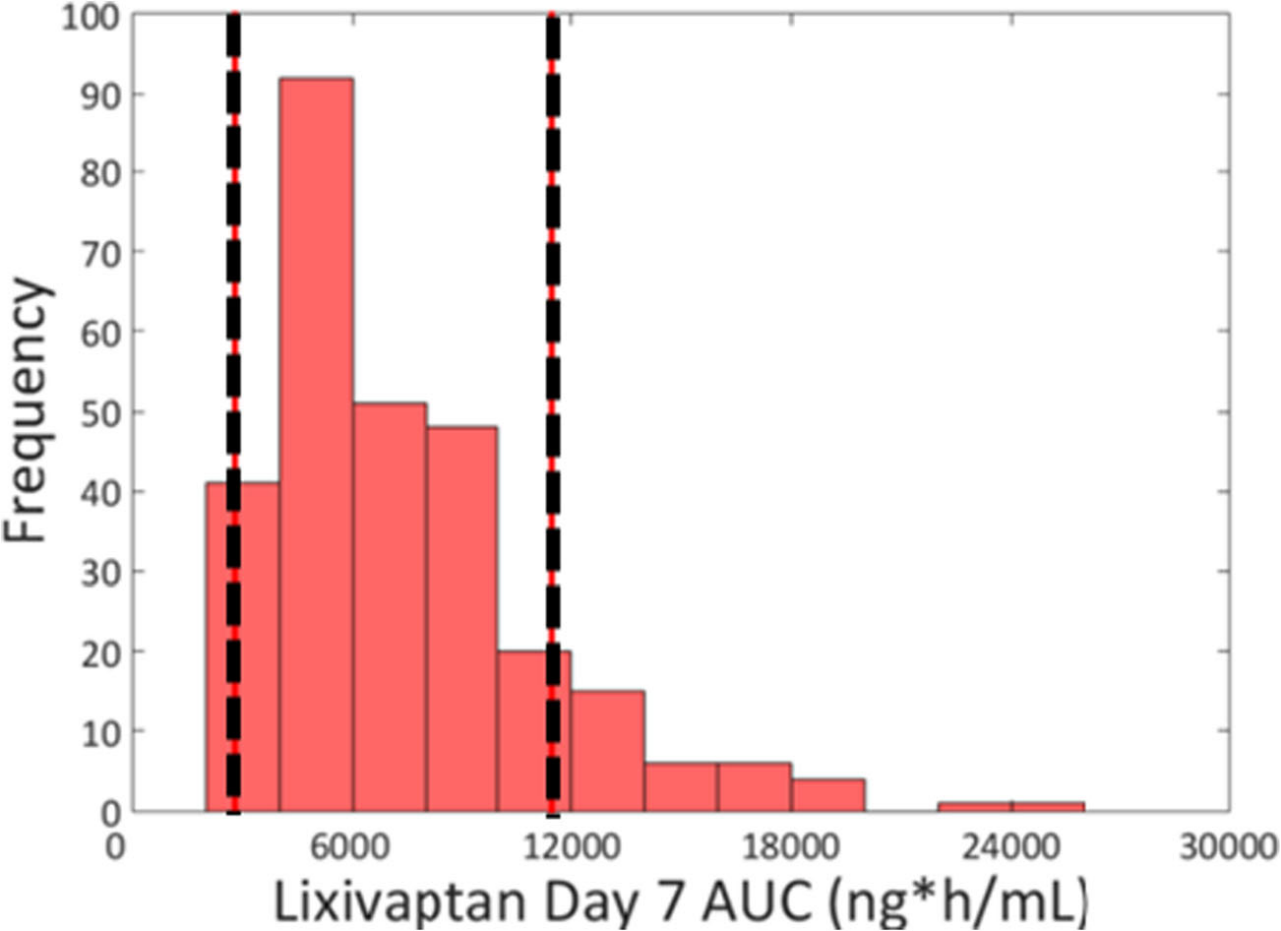
Distribution of Day 7 plasma AUC values for lixivaptan from the customized SimPops after 100 mg BID dosing for 7 days compared to the maximum and minimum (dashed lines) Day 7 AUC observed in clinical study CK-LX1403.

As part of the development program for hyponatremia, lixivaptan was dosed in about 1700 subjects across 36 clinical studies (1). The PBPK modeling was successful in recapitulating the clinically observed plasma time courses of lixivaptan and its metabolites, as well as the likely liver-to-plasma ratio for the molecules based on rat mass balance studies (data not shown) and on data collected for this work. The results for the baseline lixivaptan model are shown in Fig. 1; further information on the PBPK model can be found in Supplement A. The custom SimPops also was successful in representing the range of plasma concentrations observed in the clinic; the range of lixivaptan plasma time courses compared to the maximum and minimum observed clinical exposures is shown in Fig. 2, while a histogram of plasma AUC values compared to the maximum and minimum clinically observed plasma AUC is shown in Fig. 3. The relationship between the simulated and clinically observed AUC and C_max_ for lixivaptan and its metabolites after 7 days of 100 mg BID dosing is shown in Table 1. Further details of the PBPK modeling results, including plasma time course results and SimPops construction results for the metabolites, are given in Supplement A.

**Table 1:** Ratio of simulated vs. clinically-observed pharmacokinetic parameters for lixivaptan and its metabolites after 100 mg BID dosing for 7 days. AUC is calculated for Day 7

Chemical Species	Simulated / Observed		Liver:Plasma Ratio
	Day 7 C_max_	Day 7 AUC	
Lixivaptan	1.45	1.45	13.5*
WAY-138451	1.13	1.02	
WAY-141624	0.94	0.97	
WAY-138758	0.93	0.97	

*Intracellular concentration measurements from *in vitro* studies and rat WBAR data both suggested a value of around 17; 13.5 was close and generated a reasonable fit with the remainder of the data

### *In Vitro* Assay Results

The *in vitro* assay results suggest the theoretical potential for lixivaptan and/or its metabolites to inhibit bile acid transporters, induce oxidative stress, and/or induce mitochondrial toxicity in a dose-dependent manner *in vivo*. In addition, the *in vitro* assay results for the comparator drug tolvaptan obtained in these experiments demonstrate good correspondence with previously published results (9), confirming the validity of the comparison between simulated lixivaptan and previously-published results about tolvaptan. The results of these assays can be found in Supplement B.

### Translating *in Vitro* Assay Data to DILIsym Parameter Values

For the simulations of lixivaptan and its metabolites in DILIsym, the *in vitro* assay data were translated to DILIsym parameter values. These parameters are not assessments of toxicity risk in and of themselves; they have meaning only within the context of the DILIsym representation for each compound. The method of translation varies by the type of data available and the corresponding mechanism in DILIsym. The following sections detail this translation for bile acid transporter inhibition, mitochondrial toxicity, and oxidative stress. DILIsym parameter values for lixivaptan and its metabolites are summarized in Table 2. A DILIsym ETC inhibition parameter value was also determined for tolvaptan for the purpose of comparing the ETC inhibition observed in the current work to the previously published result (9).

**Table 2:** DILIsym simulation input parameters calculated from *in vitro* data for lixivaptan and its three metabolites. These parameters were used in the DILIsym predictions of the toxicity risk for lixivaptan. For the RNS/ROS production rate constants and NTCP inhibition constants, higher values are associated with higher risk. For all other parameters, lower values are associated with higher risk

Mechanism	DILIsym Parameter	Unit	Value***			
			Lixivaptan	WAY-138451	WAY-141624	WAY-138758
Mitochondrial Dysfunction	Coefficient for ETC inhibition	μM	535	250	N/A	N/A
Oxidative Stress	RNS/ROS production rate constant	mL/nmol/h	5.45 × 10^−4^	2.12 × 10^−3^	N/A	N/A
Bile Acid Transporter Inhibition	BSEP inhibition constant	μM	15*	8.6*	39.5*	5.6*
	NTCP inhibition constant	μM	19*	N/A	85.8*	8.9*
	Basolateral inhibition constant**	μM	70*	54*	16.3*	4*

*IC_50_ values; default assumption is mixed inhibition type with α = 5, based on the authors’ experience

**Basolateral inhibition constant represents the lowest IC_50_ of the experimentally derived MRP3 and MRP4 IC_50_ values

***Values shown in the table for DILIsym input parameters should not be interpreted in isolation with respect to clinical implications. Their predictive value resides in the combination with exposure in the context of a DILIsym simulation

#### Bile Acid Transporter Inhibition by Lixivaptan and its Metabolites

The estimated IC_50_ values for lixivaptan and its metabolites listed in Table 2 were used as the K_i_ values in DILIsym; while IC_50_ and K_i_ values can differ, this approximation is reasonable when the assay substrate concentration is well below the K_m_ for the transporter, as is the case for these studies. Because K_i_ studies were not performed for the bile acid transporters, mixed inhibition with alpha of 5 was assumed for all transporters, as described in the Methods section. This assumption was validated by sensitivity analyses simulations conducted to determine if the mode of bile acid transporter inhibition was an important factor, as also described in the methods section.

#### Mitochondrial Toxicity Parameters for Tolvaptan, Lixivaptan, and WAY-138451

**Fig. 4:**
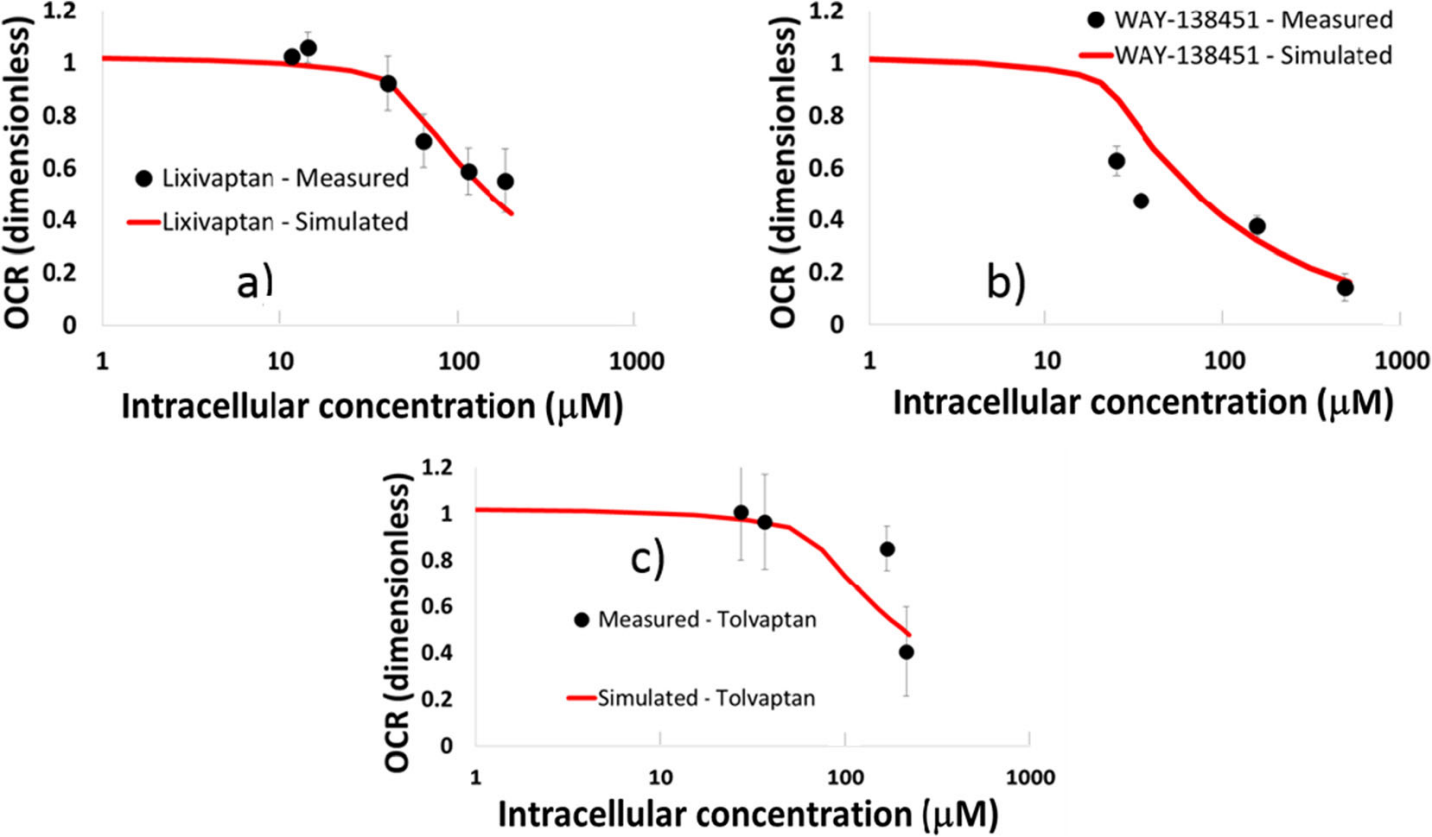
MITOsym simulations used to calculate ETC inhibition parameters for (a) lixivaptan; (b) its WAY-138451 metabolite; and (c) tolvaptan, compared with measured 24 h HepG2 data.

To define the DILIsym parameter values for tolvaptan-, lixivaptan-, and WAY-138451-mediated mitochondrial toxicity, the 24 h *in vitro* data were simulated within MITOsym (Fig. 4) and subsequently translated to DILIsym values as described in the Methods section. The intracellular concentration of tolvaptan, lixivaptan, and WAY-138451 at each dose was measured by LC/MS/MS analysis and was employed in parameterization. Reproduction of each compound’s inhibition of mitochondrial respiration in DILIsym defined them all as potential ETC inhibitors. The calculated DILIsym parameter of 729 μM for tolvaptan for this work is 35% smaller than the parameter used in previously published research, which was 1.09 mM (9). The difference is likely due to the fact that intracellular concentrations are available for this work while they were not measured for prior published work (9). This more potent ETC inhibition coefficient would likely have led to an increased incidence of simulated ALT elevations for tolvaptan had it been used in the published work. Calculated parameters for lixivaptan (represented by ETC inhibition 1) and WAY-138451 (represented by ETC inhibition 2) are listed in Table 2; they are both predicted to be mildly more potent inhibitors than tolvaptan. WAY-141624 and WAY-138758 had no effect on mitochondrial respiration (Supplement B).

#### Oxidative Stress Due to Lixivaptan and WAY-138451

**Fig. 5:**
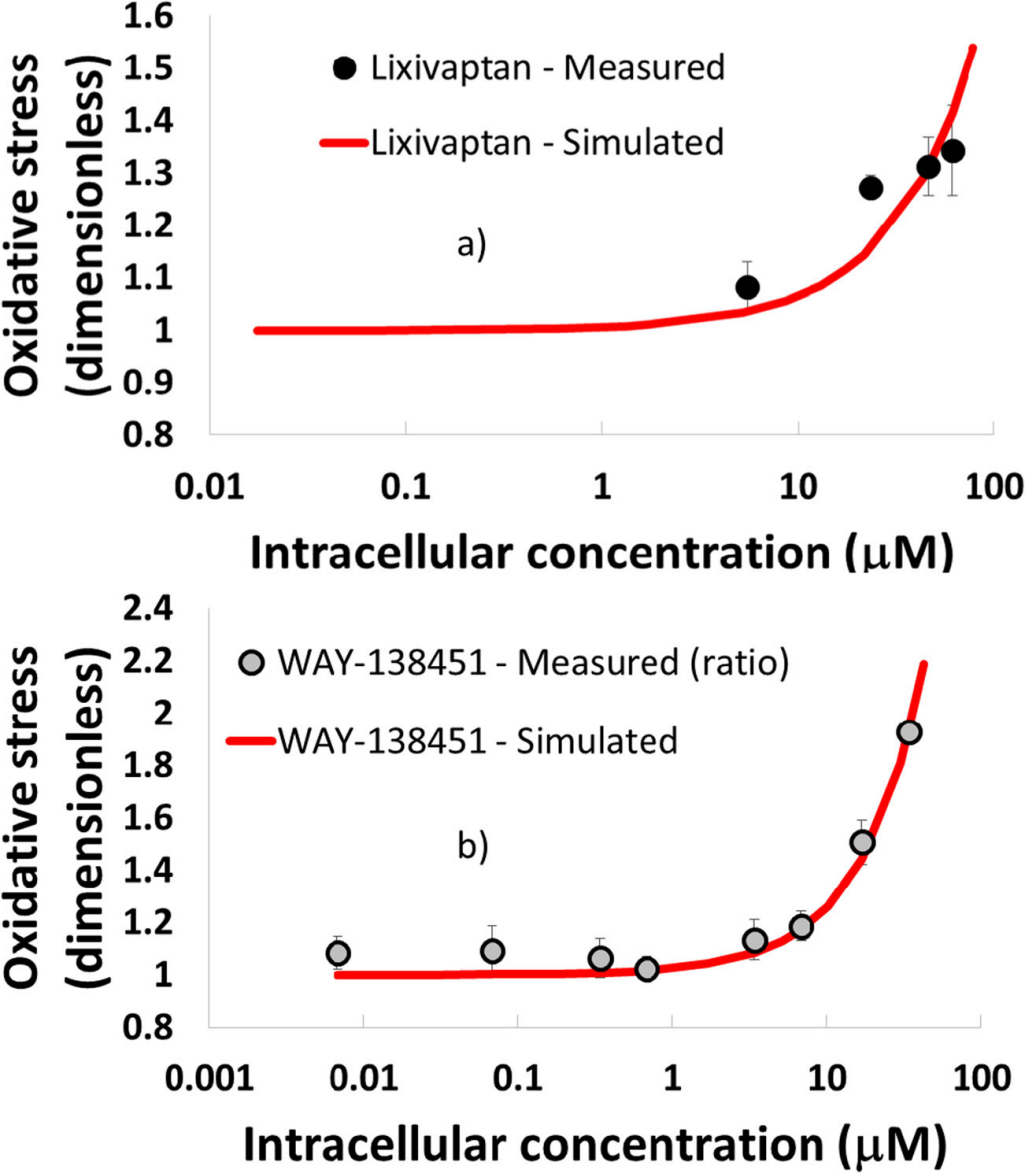
DILIsym simulations used to calculate ROS generation parameters for a) lixivaptan; and b) its WAY-138451 metabolite, compared with measured 24 h HepG2 data. For WAY-138451, the gray dots denote intracellular concentrations calculated by extrapolating the average measured ratio between nominal media and intracellular concentration for the two highest WAY-138451 concentrations to all concentrations for which ROS was measured.

To define the DILIsym parameter values for lixivaptan- and WAY-138451-mediated oxidative stress, the 24 h *in vitro* data were simulated within DILIsym (Fig. 5). The intracellular concentration of each compound was measured by LC/MS/MS analysis. For lixivaptan, the measured intracellular concentration at each dose was used to parameterize the model. For WAY-138451, however, the LC/MS/MS data was inconsistent at low doses due to lower limit of detection issues. Thus, the mean measured cell-to-nominal-media ratio (0.672) was used to estimate intracellular concentrations at each dose. Reproduction of lixivaptan- and WAY-138451-mediated induction of oxidative stress defined the relationship between liver compound concentration and ROS formation with the reactive nitrogen/oxygen species (RNS/ROS) production rate constants listed in Table 2. WAY-141624 and WAY-138758 had no effect on cellular oxidative stress.

### Toxicity Simulation Results

Simulations were conducted in the customized SimCohorts and SimPops to characterize the potential for lixivaptan-mediated mitochondrial dysfunction, oxidative stress, and bile acid transporter inhibition to induce hepatotoxicity.

#### Toxicity Simulations in SimCohorts Not Including Exposure-Related Variability

A series of simulations using the Multi16 SimCohorts were performed on the completed and proposed clinical protocols listed in the Methods section. These results are presented in Table 3. Notably, the results for the 200/100 split daily dosing protocol were unchanged over the 12-week simulation period using alternate bile acid transporter modes of inhibition, i.e., the default assumption (all transporters mixed with α = 5) to the “worst-case scenario” (basolateral and canalicular efflux transporters noncompetitive, uptake transporters competitive); in both cases no toxicity was predicted. Therefore IC_50_ values with mixed inhibition, α = 5, were used for subsequent SimPops simulations. As a result of these initial sensitivity analysis simulations, further experiments to determine the mode of bile acid transporter inhibition were not conducted.

**Table 3:** Simulation results for lixivaptan from the Human_ROS_apop_mito_v4A_1_Multi16 SimCohorts, a group of simulated individuals (*n* = 16) among the full v4A_1 SimPops (*n* = 285) included in DILIsym v6A; 13 of the simulated individuals are sensitive to DILI mechanisms or combinations, 2 are insensitive, and 1 is the baseline (average) human. This SimCohorts does not include lixivaptan exposure-related parameter variability

Dose	Duration	Clinical Protocol	Parameter Settings	ALT >2X ULN*	ALT >3X ULN*
100 mg BID	60 days	Hyponatremia	Default measured^#^	0/16	0/16
200 / 100 mg	12 weeks	ADPKD	Default measured^#^	0/16	0/16
200 / 100 mg	12 weeks	ADPKD	BA uptake competitive, BA efflux non-competitive^##^	0/16	0/16
400 mg BID	7 days	Supratherapeutic dose	Default measured^#^	1/16	0/16

*Upper limit of normal (ULN) in DILIsym is 40 U/L

^#^Default assumption for bile acid (BA) inhibition is mixed inhibition type with α = 5 in the absence of Ki studies, based on the authors’ experience

^##^This is the most conservative assumption from a safety standpoint and helps determine if Ki studies are needed. Mixed inhibition is set to noncompetitive for BA efflux and competitive for BA uptake

#### Toxicity Simulations in SimPops with Exposure-Related Variability

**Fig. 6:**
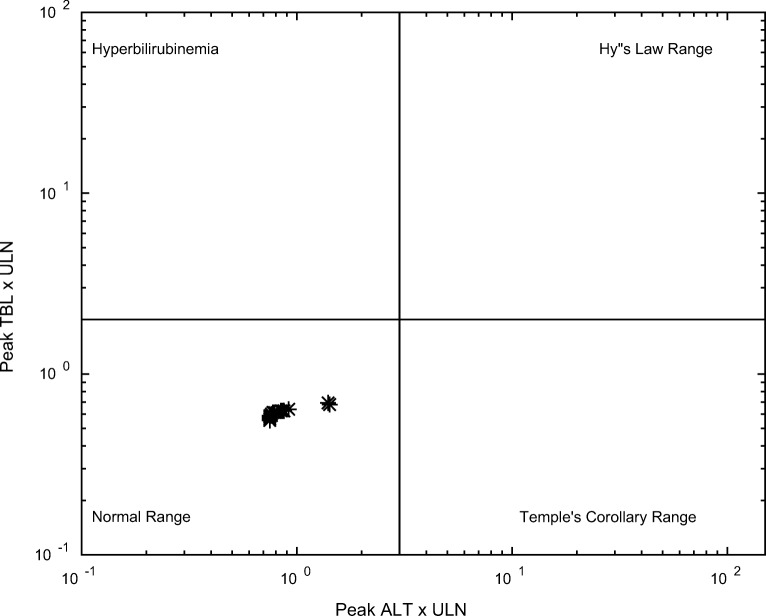
eDISH (evaluation of Drug-Induced Serious Hepatotoxicity) plot showing DILIsym simulated liver safety outcomes for 200/100 mg split daily dosing of lixivaptan over 12 weeks in the lixivaptan-specific SimPops of 285 simulated normal healthy volunteers including lixivaptan PK variability.

Results from simulations using the full 285-individual lixivaptan-specific SimPops are shown in Table 4. No clinically significant ALT elevations (defined as >3X ULN) were observed in the 100 mg BID 60-day simulation, consistent with clinical observations that found no difference in liver signals between lixivaptan and placebo at this dose (13). When the proposed clinical dosing protocol of 200/100 mg split daily dosing for the treatment of ADPKD was simulated over 12 weeks, no ALT elevations greater than 2X ULN were observed; the highest observed ALT value was 57 U/L, corresponding to 1.4 X ULN. This suggests that no clinically-significant ALT elevations would be expected if this dose were used in clinical trials. Fig. 6 shows the simulated eDISH plot (15) for the proposed clinical protocol of 200/100 mg split daily doses of lixivaptan.

**Table 4:** Simulation results for lixivaptan from the custom 285-individual SimPops including variability in lixivaptan exposure-related parameters

Dose	Duration	Clinical Protocol	Parameter Settings	Clinical ALT >3X ULN	Simulated ALT >3X ULN*
100 mg BID	60 days	Hyponatremia	Default measured^#^	On treatment similar to placebo**	0/285
200 / 100 mg	12 weeks	ADPKD	Default measured^#^	Clinical study not yet conducted	0/285
400 mg BID	7 days	Supratherapeutic dose	Default measured^#^	0/67	7/285

*Upper limit of normal (ULN) in DILIsym is 40 U/L

**Data from study CK-LX3401 (not shown)

^#^Default assumption for BA inhibition is mixed inhibition type with α = 5 in the absence of K_i_ studies, based on the authors’ experience

At supratherapeutic doses of 400 mg BID for 7 days, 7/285 (2.46%) of simulated individuals had an ALT >3X ULN, whereas no individuals out of 67 showed elevated ALT in the CK-LX1403 clinical trial. The simulation result is not out of the realm of possibility, but suggests that the DILIsym simulation for lixivaptan yields results that are likely conservative compared to the clinical experience. Of note, the ALT elevations seen at the 400 mg BID dose in the DILIsym simulation were mild and were not associated with signs of severe hepatocellular toxicity.

#### Mechanistic Investigation Simulations

Results from simulations intended to explore the mechanisms of simulated ALT elevations with lixivaptan are reported in Table 5; the full Lixivaptan-Specific SimPops was used for these simulations. Changing the bile acid transporter mode of inhibition had no effect on the predictions, suggesting that bile acid transport is not a mechanism likely to contribute to liver signals with lixivaptan. Conversely, when the contribution of lixivaptan-induced ROS formation was turned off in the simulation, no ALT elevations were seen at the simulated 400 mg BID dose or at the 800/400 mg dose, i.e. doses 4 times higher than the proposed maximum clinical dose for ADPKD. Importantly, unlike the case of tolvaptan and its major accumulating metabolite DM-4103 (9), the metabolites of lixivaptan did not contribute meaningfully to the ALT elevation signal; the entirety of the observed signals can be explained by the parent. The results from these simulations suggest that ROS production by the parent compound is the key contributing mechanism to the ALT elevation observed in the simulation at supratherapeutic doses.

**Table 5:** Lixivaptan simulation results from simulations performed for the purpose of mechanistic exploration in both the custom 285-individual SimPops and the Lixivaptan-Sensitive SimCohorts, a 16-individual SimCohorts of sensitive individuals taken from the custom lixivaptan SimPops

Dose	Duration	Parameter Settings	SimPops or SimCohorts Used	Simulated ALT >3X ULN*
200/100 mg	12 weeks	Worst-case BA inhibition scenario^##^	Full customized SimPops	0/285
400 mg BID	7 days	Default measured^#^	Full customized SimPops	7/285
400 mg BID	7 days	No parent-generated ROS	Full customized SimPops	0/285
800/400 mg	12 weeks	No ROS generation by parent or metabolites	Lixivaptan-Sensitive Simcohorts	0/16

*Upper limit of normal (ULN) in DILIsym is 40 U/L

^#^Default assumption for BA inhibition is mixed inhibition type with α = 5 in the absence of Ki studies, based on the authors’ experience

^# #^Worst-case BA inhibition scenario is competitive NTCP inhibition and non-competitive basolateral and canalicular inhibition

#### Dose Escalation Simulations

Results from simulated dose escalation studies performed on the Lixivaptan-Sensitive SimCohorts, which includes a broad range of exposure-related and susceptibility-related variability, are listed in Table 6. Simulated clinically significant (> 3X ULN) ALT elevations began to occur at 1.5X above the proposed maximum clinical dose of 200/100 mg split daily dosing. However, even in this sensitive population, severe liver injury, as defined by Hy’s Law criteria (ALT >3X ULN, bilirubin >2X ULN), was not observed until doses of 600/300 mg BID, i.e. 3X above the proposed maximum clinical dose.

**Table 6:** Lixivaptan dose-escalation simulation results performed in the Lixivaptan-Sensitive SimCohorts, a custom SimCohorts enriched with 16 toxicity-sensitive individuals taken from the custom lixivaptan SimPops, for 12 weeks. Dose escalation was conducted on this sensitive population to amplify a potential signal without excessive computational cost

Dose	Duration	Parameter Settings	Simulated Individuals with ALT >3X ULN*
200/100 mg	12 weeks	Default measured^#^	0
300/150 mg	12 weeks	Default measured^#^	2
400/200 mg	12 weeks	Default measured^#^	6
500/250 mg	12 weeks	Default measured^#^	8
600/300 mg	12 weeks	Default measured^#^	10

*Upper limit of normal (ULN) in DILIsym is 40 U/L

^#^Default assumption for BA inhibition is mixed inhibition type with α = 5 in the absence of K_i_ studies, based on the authors’ experience

#### Simulated Exposure-Response Relationship

**Fig. 7:**
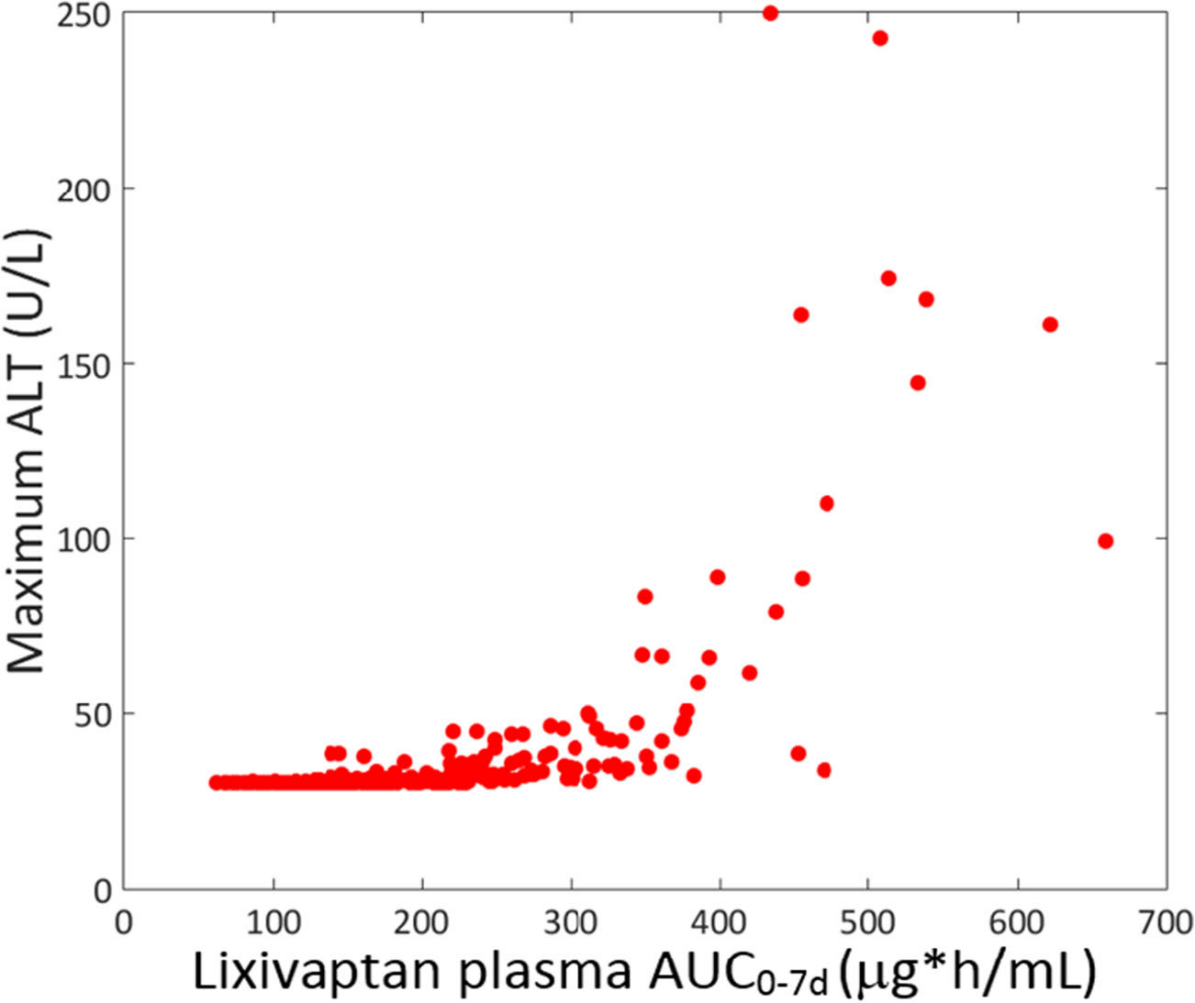
Relationship between simulated peak ALT value and simulated AUC_0-inf_ for lixivaptan simulations conducted at a supratherapeutic dose of 400 mg BID for 7 days. Upper limit of normal (ULN) for ALT is 40 U/L in DILIsym.

Of particular interest in the Lixivaptan-Specific SimPops simulation results for the supratherapeutic 400 mg BID dose is the relationship between exposure and ALT elevation, as shown in Fig. 7. Every simulated individual who developed an ALT elevation at 400 mg BID dosing had a lixivaptan AUC_0–7 day_ greater than 300 μg·h/mL; the results demonstrate a clear relationship between simulated exposure and simulated response.

## Discussion and Conclusions

The DILIsym simulations presented here suggest that the second-generation vasopressin V2 receptor antagonist lixivaptan is not likely to cause liver injury due to the mechanisms included in DILIsym v6A. DILIsym successfully recapitulated the observed clinical data regarding the safety of lixivaptan given at 100 mg BID for 60 days. Furthermore, simulation results for lixivaptan predicted no ALT elevations at the proposed clinical dose of 200/100 mg BID for ADPKD. This result compares favorably to the results previously published for tolvaptan at the FDA-approved dose of 90/30 mg split daily dosing for ADPKD (9); this comparison is shown in Table 7. An ALT >3X ULN elevation frequency of 7.86% was predicted for tolvaptan (compared to an observed frequency range of 4.4% to 5.6% in the clinic (2, add REPRISE study citation)). As no elevations were predicted for lixivaptan, the simulation results suggest that lixivaptan is likely safer than tolvaptan with respect to the liver toxicity mechanisms included in DILIsym v6A. Although lixivaptan and metabolites also show the potential to interact with bile acid transporters, the predicted difference in toxicity between lixivaptan and tolvaptan is due, in large part, to higher liver concentrations predicted for tolvaptan compared to lixivaptan, particularly for the molecular entities that potently interact with bile acid transporters.

**Table 7:** Comparison between simulation and clinical results for lixivaptan from this study and for tolvaptan from previously published research (9) at the maximum intended doses for ADPKD

Drug	Dose	Duration	Parameter Settings	Simulated ALT >3X ULN*	Clinical ALT >3X ULN	Simulated Hy’s Law Cases	Clinical Hy’s Law Cases
Lixivaptan	200/100 mg	12 weeks	Default measured^#^	0/285 (0.0%)	Study not yet conducted	No	Study not yet conducted
Tolvaptan	90/30 mg	24 weeks	Default measured^#^	18/229 (7.86%)	4.4% and 5.6%	Yes	Yes

*Upper limit of normal (ULN) in DILIsym is 40 U/L

^#^Default lixivaptan assumption for BA inhibition is mixed inhibition type with α = 5 in the absence of K_i_ studies, based on the authors’ experience

DILIsym simulations predicted rare ALT elevations with lixivaptan at supratherapeutic doses of 400 mg BID for 7 days, whereas no elevations were observed at this dose in clinical studies. This prediction is not unreasonable, considering the larger size of the DILIsym simulated population compared to the clinical trial population (285 simulated individuals vs. 67 treated subjects in the clinical study); however, it also suggests that DILIsym’s predictions for lixivaptan tend to overstate the clinical toxicity and therefore are to be considered conservative. This should provide added confidence in the results suggesting that lixivaptan is not likely to cause liver injury.

Interestingly, mechanistic analyses suggested that lixivaptan’s primary toxicity mechanism of relevance (at simulated supratherapeutic doses) is likely to be ROS generation, whereas simulated tolvaptan toxicity was predicted to be a product of both bile acid accumulation and mitochondrial ETC inhibition. This further suggests substantial qualitative differences between lixivaptan and tolvaptan in terms of their likely liver effects. Such differences could potentially play a meaningful role in the differential clinical safety profile of the two drugs. First, drugs that have the potential to cause hepatocellular dysfunction through two or more concurrent mechanisms, like tolvaptan, have been associated with particularly pronounced liver injury (16). Second, new evidence suggests that ADPKD patients may have altered mitochondrial function and bile acid transport compared to healthy individuals, which could render them more susceptible to the mechanisms of hepatocellular injury associated with tolvaptan (17–19). Lastly, ALT elevations that are induced by ROS formation are generally prone to resolution on treatment (20), suggesting that any potential ALT elevation observed with lixivaptan in the clinics may be self-limiting.

The full SimPops simulation of lixivaptan was performed for 12 weeks in 285 simulated individuals, while the published simulations for tolvaptan were for 24 weeks in 229 simulated individuals (7). Based on analysis of mechanistic endpoints in the 285-individual results for lixivaptan, it is unlikely that longer simulations would lead to ALT elevations for lixivaptan as occurred for tolvaptan. More specifically, bile acid concentrations and ETC activity had achieved steady state across the SimPops by 96 h in the lixivaptan simulations (data not shown), suggesting that toxicity would not manifest itself with longer simulation times. In order to further validate this assumption, a 24-week simulation was performed in the customized 16-individual Lixivaptan-Sensitive SimCohorts. No difference was observed between the 12-week simulation and the 24-week simulation (data not shown). Therefore, the simulation time difference is unlikely to impact the conclusions of this project and, by extension, the conclusions are likely applicable to the chronic use of lixivaptan. This is in striking contrast with tolvaptan, for which toxicity parameters continue to increase over time in the DILIsym simulation, closely mimicking the clinical observation that liver toxicity can take up to 18 months to manifest itself (2).

Another interesting point of comparison between lixivaptan and tolvaptan is the role of exposure in the simulated toxicity. For tolvaptan, exposure was not observed to be related to toxicity (2); this was also the case in the simulation results, where exposure-related parameters were not directly correlated to ALT elevations (9). Conversely, when toxicity was simulated to occur with supra-therapeutic doses of lixivaptan, it was predicted to be directly exposure-related; this makes the manifestation of rare toxicity at lower doses much less likely than was the case with tolvaptan and provides an avenue to inform dosing decisions. In particular, doses of lixivaptan such as the proposed maximum dose for ADPKD of 200/100 mg lead to drug exposures well below the threshold for safety, as shown in Fig. 6, and are therefore expected to be safe. The difference in the exposure-response relationship between the two drugs is likely due to the difference in simulated toxicity mechanisms for the two molecules; the relationship between ROS and cell death in DILIsym is more direct (10), while bile acid accumulation and mitochondrial dysfunction can be heavily influenced by individual susceptibility factors (6,21).

It has been proposed that an adaptive immune-mediated attack plays a role in tolvaptan-mediated toxicity (2). DILIsym v6A does not represent the adaptive immune system and, as such, cannot determine with absolute certainty that an adaptive immune mechanism would not play a role in the case of lixivaptan as well. However, tolvaptan-mediated injury has been shown to correlate with biomarkers of disrupted bile acid homeostasis in a mouse model (22), suggesting that the mechanisms tested in DILIsym are significant contributors to the observed toxicity. Furthermore, it has been theorized that an underlying cellular stress response is a necessary but not sufficient step in an adaptive immune DILI attack (23,24); the DILIsym results show that this cellular stress is less likely to manifest with lixivaptan than with tolvaptan, implying that the former would be more likely to be safe clinically.

It is also important to understand the potential effects of uncertainty in the model on the predictions generated here. In Table 6, the dose of lixivaptan was increased by 50%, which is well above the expected uncertainty in the liver concentration as determined by the variance between simulated and observed PK parameters in the PBPK model. These simulations were performed in individuals that were more sensitive to lixivaptan and, per Fig. 7, had a higher exposure to lixivaptan. The fact that only 2 simulated individuals developed mild ALT elevations after this dose escalation suggests that uncertainty in the PBPK model likely did not influence the conclusions from this work.

In sum, DILIsym simulation results suggest that lixivaptan is less likely to cause hepatotoxicity than tolvaptan at their respective therapeutic doses for the treatment of ADPKD. These results bode well for the continued development of lixivaptan as a safe therapy for the treatment of this disease. Finally, this work illustrates the applicability of QST modeling methods in comparing the toxicity potential of compounds within the same class. The difference in likely toxicity mechanisms between tolvaptan and lixivaptan demonstrates the fact that compounds within the same class can vary widely in terms of potential liability and potential mechanisms of toxicity.

## Electronic supplementary material

ESM 1 (DOCX 639 kb)

ESM 2 (DOCX 1221 kb)

ESM 3 (MAT 8 kb)

ESM 4 (MAT 35 kb)

ESM 5 (MAT 77 kb)
