# Supplementary material for: Comparison of the Hepatotoxic Potential of Two Treatments for Autosomal-Dominant Polycystic Kidney DiseaseUsing Quantitative Systems Toxicology Modeling
Source: Pharm Res. 2020 Jan 6;37(2):24. doi: 10.1007/s11095-019-2726-0 (PMC6944674; doi:10.1007/s11095-019-2726-0)
Supplement: Supplementary file 1 — (DOCX 639 kb) [file 11095_2019_2726_MOESM1_ESM.docx]

# Supplement A: PBPK Modeling and Custom SimPops Creation for Lixivaptan and Its Metabolites

## PBPK modeling methods

A PBPK representation for lixivaptan and its three major metabolites, WAY-138451, WAY-141624, and WAY-138758, was constructed within DILIsym for this work. The PBPK representation of lixivaptan was based on the following data: Clinical study CK-LX1403 (100 mg and 400 mg BID dosing for 7 days in healthy volunteers; N = 75 and 69, respectively) and clinical study 0892A1-109-US (100 mg single-dose mass balance study in healthy volunteers) (data not shown). Furthermore, a rat tissue distribution study and a CYP450 metabolism study (data not shown) were used to help identify PBPK parameters in DILIsym. The full list of PBPK parameters, including those optimized and those derived from experimental data, are listed in Table A1.

For lixivaptan, data only minimally supported transporter-mediated hepatic transport (proprietary data not shown); lixivaptan distribution was assumed to be perfusion-limited with high permeability into all tissues. Lixivaptan tissue distribution was represented by partition coefficients (first-order, gradient driven distribution), the ratio among which was estimated based on its physicochemical properties. In the current PBPK model, lixivaptan metabolism was represented by four metabolic pathways: WAY-138451, WAY-141624, WAY-138758, and other metabolites not contributing to toxicity. *In vivo* mass balance data indicated that lixivaptan is also excreted into urine and feces unchanged. Lixivaptan is highly bound to plasma proteins; it has a measured plasma fraction unbound in human of less than 1% and a blood:plasma ratio of 0.6 (proprietary data not shown) .

### PBPK model optimization of lixivaptan

For parameter optimization, DILIsym parameters that needed to be optimized were first identified. The lixivaptan metabolic K_m_ for all chemical species was estimated using microsomal metabolism data (proprietary data not shown). The metabolic V_max_ was optimized because V_max_ values derived from *in vitro* metabolism data could not reliably recapitulate plasma PK profiles; the general order of metabolism (i.e. WAY-138451 > WAY-141624 > WAY-138758) was the same for the optimized values as observed in the *in vitro* study. Renal and biliary clearance values were optimized for each chemical species, as was the volume of distribution for the metabolites and the partition coefficients for the parent compound (though the partition coefficients were treated as covariates, maintaining the ratio determined by the correlation to physicochemical properties). The plasma fraction unbound was optimized within the >99% range suggested by *in vitro* data. Parameters involved in intestinal absorption were also optimized. Optimization was conducted until a) the plasma time courses of lixivaptan and its metabolites were in agreement with the observed data after 7 days 100 mg BID dosing; b) the time course of lixivaptan was in agreement with observed data after 7 days 400 mg BID dosing; and c) the excretion balance for all species was in agreement with observed data following a 100 mg single dose. Agreement with data within the optimization process was defined based on minimization of the sum of squares of the difference between data and simulation results. Following optimization, model-simulated plasma AUC and plasma C_max_ were evaluated to ensure consistency with observed data.

## SimPops construction for lixivaptan

A custom SimPops including variability in parameters related to lixivaptan exposure was constructed for this work. Parameters that were likely to vary within a population were selected and assigned ranges and distributions. These ranges were assumed to be between 33% and 166% of their baseline value unless published data or the comparison between simulated and observed plasma time courses suggested otherwise. Specialized ranges were utilized for WAY-138451 biliary clearance (an expanded range was used to capture the observed variability in plasma PK) and for metabolism (range suggested by Achour 2014 (9); metabolite V_max_ values were treated as covariates for the SimPops construction). The parameter values varied in the SimPops and their attendant ranges are listed in Table A2.

285 individuals representing a range of pharmacokinetic behavior for lixivaptan and its metabolites were selected for this SimPops. Each individual lixivaptan-specific parameter set was associated randomly with a simulated individual from the Human_ROS_apop_mito_BA_v4A_1 SimPops already included in the DILIsym software. The parameters varied in this SimPops as well as the sources for their ranges of variability are listed in Table A3. The simulation size of 285 individuals selected for this project is similar to that used for the majority of DILIsym simulation work (3–5), including the simulations conducted for tolvaptan (7), and captures a sufficiently wide repertoire of individuals to allow conclusive predictions. While the population size could be larger, there is an attendant computational cost associated with their generation and qualification, as well as the simulation time for longer duration therapies (such as 12 weeks simulated for lixivaptan).

Smaller groups of individuals from the v4A_1 SimPops, called SimCohorts, are also included with DILIsym v6A. The Multi16 SimCohorts is used in this work. It consists of individuals that are sensitive to various mechanisms of toxicity, including mitochondrial dysfunction, oxidative stress, bile acid accumulation, and a combination of mitochondrial and bile acid mechanisms. It also includes the baseline individual and some non-sensitive individuals from the same SimPops.

## PBPK modeling results

Simulation results from the DILIsym PBPK representation of lixivaptan and its metabolites compared to actual plasma data after 7 days of 100 mg BID dosing are shown in Figure 1 of the main text; comparison of the lixivaptan parent PBPK simulation results to actual plasma data after 7 days of 400 mg BID dosing is shown in Figure A1. The comparison of simulated AUC and C_max_ values for lixivaptan and its metabolites to actual, measured PK parameters after 7 days of 100 mg BID dosing is shown in Table 1 in the main manuscript; comparison of the simulated fecal and renal clearances of lixivaptan and its metabolites to measured data after a single 100 mg dose is shown in Table A4. Comparison of simulated pharmacokinetic parameters to clinically observed parameters for lixivaptan after 7 days of 400 mg BID dosing is shown in Table A5. For the single-dose clearance study, WAY-138451 co-eluted with other minor metabolites in the mass spectrometry analysis; the simulated values reflect those calculated for the combination of WAY-138451 and metabolites in the “other” category. The DILIsym PBPK sub-model simulation showed a good fit to the optimization data. The simulated liver-to-plasma total C_max_ ratio of 13.5 is similar to a measured value of 17 from a rat tissue distribution study (proprietary data not shown ). The PBPK model for lixivaptan and its metabolites fits the measured data reasonably well; the clearances in the single-dose study are all overestimated and this is most likely because the single-dose study reports only 84% recovery after 7 days, which is unlikely.

## SimPops construction for lixivaptan: results

Figures A2-A4, along with Figure 2 of the main manuscript, show the customized SimPops with lixivaptan exposure variability against the 100 mg BID 7-day plasma PK data (Study CK-LX1403) for lixivaptan (Figure 2, main manuscript), WAY-138451 (Figure A2), WAY-141624 (Figure A3), and WAY-138758 (Figure A4); gray circles represent the maximum and minimum values observed among all of the individuals (N = 67) in the study, whereas each colored line represents a simulated individual in the customized SimPops. The figures demonstrate that the SimPops covers the observed exposure range for each of the compounds of interest.

Figures A5-A7, along with Figure 3 of the main manuscript, show the distribution of the Day 7 plasma AUC for lixivaptan (Figure 3, main manuscript), WAY-138451 (Figure A5), WAY-141624 (Figure A6), and WAY-138758 (Figure A7). The vertical lines in the figures represent the maximum and minimum AUC observed among all the individuals in Study CK-LX1403. The figures again demonstrate that the SimPops covers the range of observed exposures. The presence of AUC values beyond the bounds of those observed in the clinical study represents the inclusion in the SimPops of outlier individuals who are plausible in a wider population but who may not occur in a study with N = 67.

## References

1. Achour B, Barber J, Rostami-Hodjegan A. Expression of hepatic drug-metabolizing cytochrome p450 enzymes and their intercorrelations: a meta-analysis. Drug Metab Dispos Biol Fate Chem. 2014 Aug;42(8):1349–56.

2. Yang K, Woodhead JL, Watkins PB, Howell BA, Brouwer KL. Systems Pharmacology Modeling Predicts Delayed Presentation and Species Differences in Bile Acid-Mediated Troglitazone Hepatotoxicity. Clin Pharmacol Ther. 2014 Jul 28;589–98.

3. Longo DM, Yang Y, Watkins PB, Howell BA, Siler SQ. Elucidating Differences in the Hepatotoxic Potential of Tolcapone and Entacapone With DILIsym(®), a Mechanistic Model of Drug-Induced Liver Injury. CPT Pharmacomet Syst Pharmacol. 2016 Jan;5(1):31–9.

4. Woodhead JL, Yang K, Siler SQ, Watkins PB, Brouwer KLR, Barton HA, et al. Exploring BSEP inhibition-mediated toxicity with a mechanistic model of drug-induced liver injury. Front Pharmacol. 2014;5:240.

5. Woodhead JL, Brock WJ, Roth SE, Shoaf SE, Brouwer KLR, Church R, et al. Application of a Mechanistic Model to Evaluate Putative Mechanisms of Tolvaptan Drug-Induced Liver Injury and Identify Patient Susceptibility Factors. Toxicol Sci Off J Soc Toxicol. 2016 Sep 21;

6. Allen JW, Shanker G, Aschner M. Methylmercury inhibits the in vitro uptake of the glutathione precursor, cystine, in astrocytes, but not in neurons. Brain Res. 2001 Mar 9;894(1):131–40.

7. Lee K-T, Tsai S-M, Wang S-N, Lin S-K, Wu S-H, Chuang S-C, et al. Glutathione status in the blood and tissues of patients with virus-originated hepatocellular carcinoma. Clin Biochem. 2007 Oct;40(15):1157–62.

8. Nagasaka H, Takayanagi M, Tsukahara H. Children’s toxicology from bench to bed--Liver Injury (3): Oxidative stress and anti-oxidant systems in liver of patients with Wilson disease. J Toxicol Sci. 2009;34 Suppl 2:SP229-236.

9. Shon Y-H, Nam K-S. Protective effect of moutan cortex extract on acetaminophen-induced cytotoxicity in human Chang liver cells. Biol Pharm Bull. 2002 Nov;25(11):1427–31.

10. Pérez-Carreras M, Del Hoyo P, Martín M a, Rubio JC, Martín A, Castellano G, et al. Defective hepatic mitochondrial respiratory chain in patients with nonalcoholic steatohepatitis. Hepatol Baltim Md. 2003 Oct;38(4):999–1007.

11. Bantel H, Ruck P, Gregor M, Schulze-Osthoff K. Detection of elevated caspase activation and early apoptosis in liver diseases. Eur J Cell Biol. 2001 Mar;80(3):230–9.

12. Meier Y, Pauli-Magnus C, Zanger UM, Klein K, Schaeffeler E, Nussler AK, et al. Interindividual variability of canalicular ATP-binding-cassette (ABC)-transporter expression in human liver. Hepatol Baltim Md. 2006 Jul;44(1):62–74.

13. Bernhardt G a, Zollner G, Cerwenka H, Kornprat P, Fickert P, Bacher H, et al. Hepatobiliary transporter expression and post-operative jaundice in patients undergoing partial hepatectomy. Liver Int Off J Int Assoc Study Liver. 2012 Jan;32(1):119–27.

Figure A1. Simulated and measured (clinical study CK-LX1403) plasma concentrations of lixivaptan after 7 days of 400 mg BID dosing.


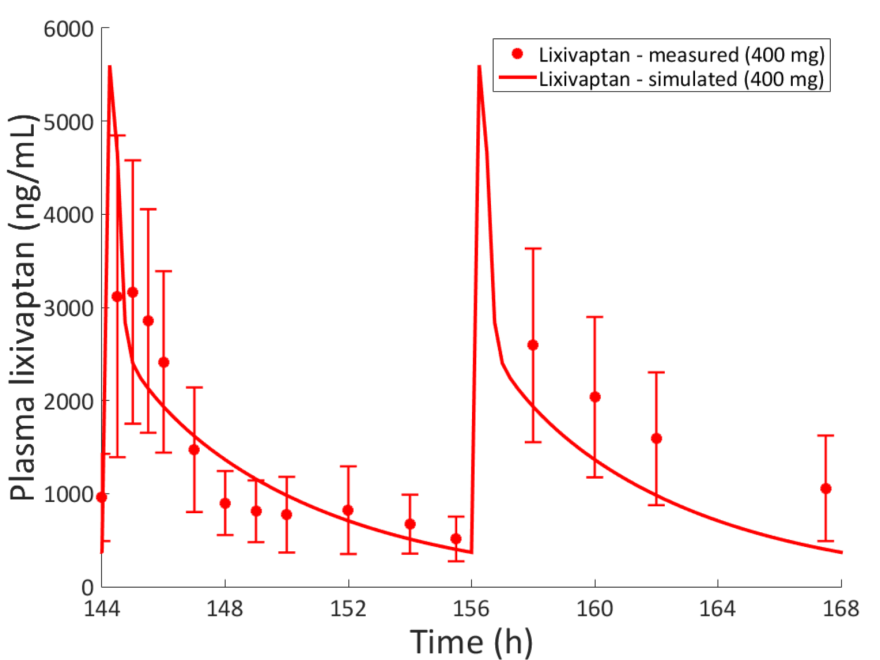


Figure A2. Simulated plasma time courses of WAY-138451 from the customized SimPops on day 7 of 100 mg BID dosing compared with the maximum and minimum concentrations measured at each time point in clinical study CK-LX1403.


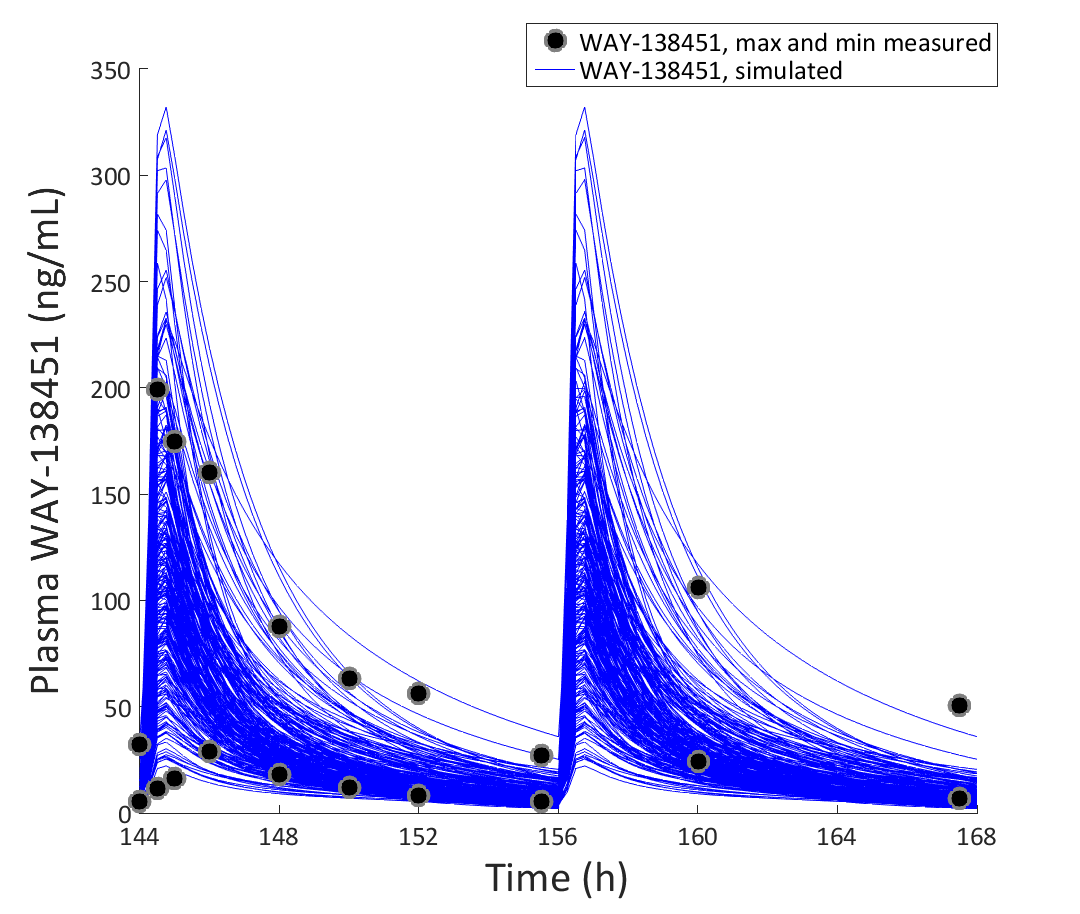


Figure A3. Simulated plasma time courses of WAY-141624 from the customized SimPops on day 7 of 100 mg BID dosing compared with the maximum and minimum concentrations measured at each time point in clinical study CK-LX1403.


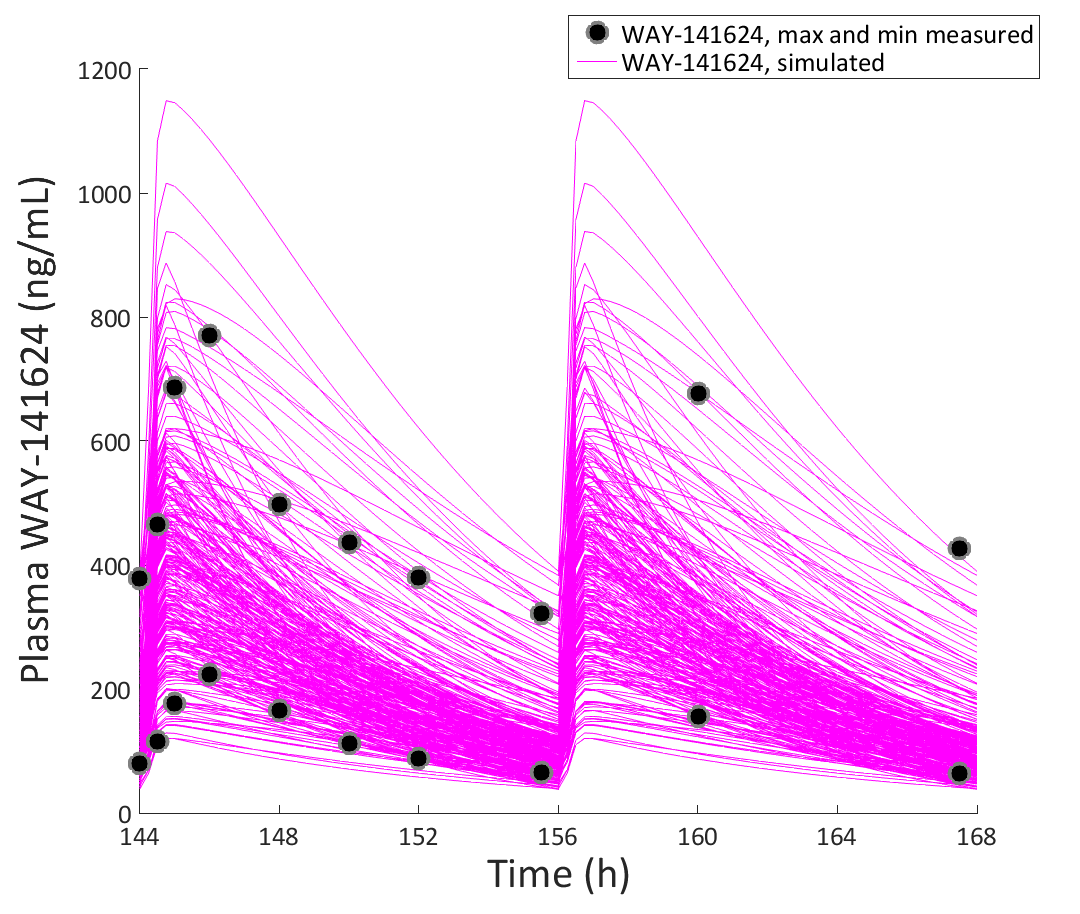


Figure A4. Simulated plasma time courses of WAY-138758 from the customized SimPops on day 7 of 100 mg BID dosing compared with the maximum and minimum concentrations measured at each time point in clinical study CK-LX1403.


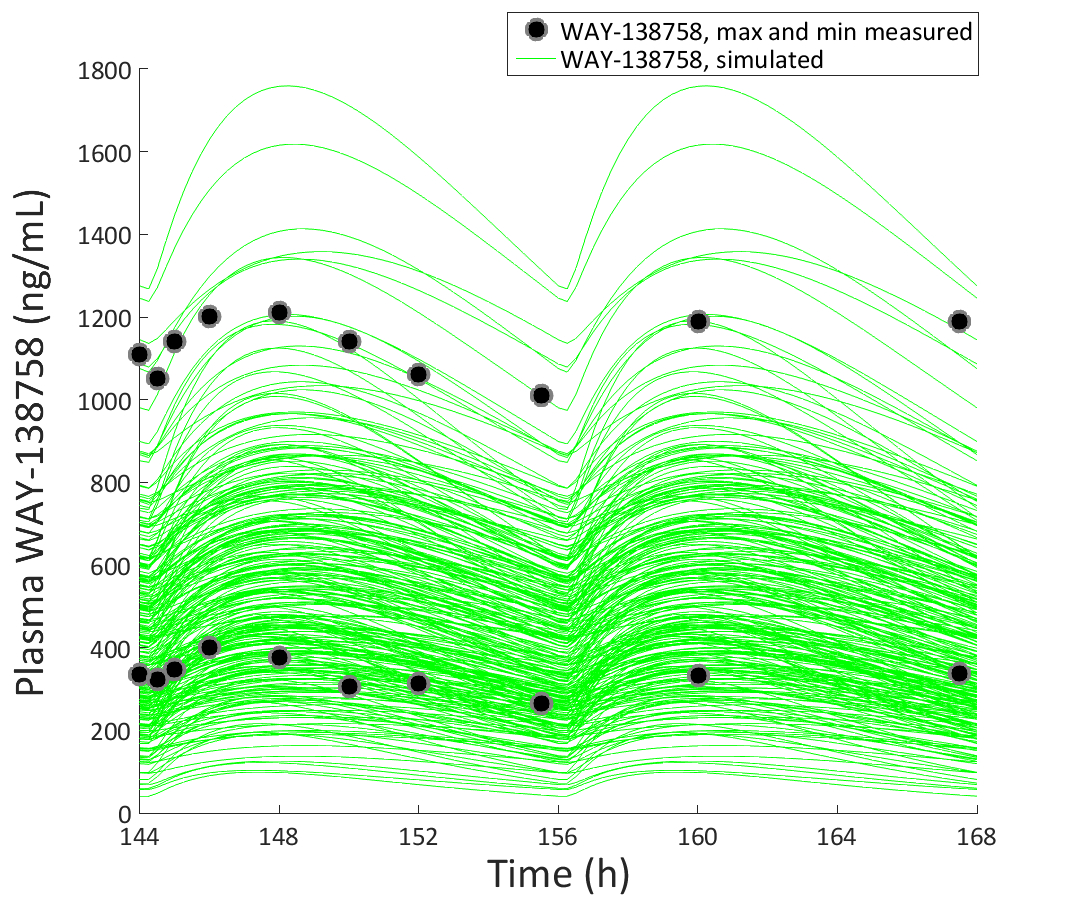


Figure A5. Distribution of Day 7 plasma AUC values for WAY-138451 from the customized SimPops after 100 mg BID dosing for 7 days compared to the maximum and minimum (dashed lines) Day 7 AUC observed in clinical study CK-LX1403.


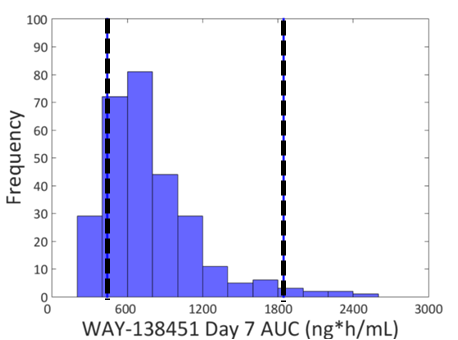


Figure A6. Distribution of Day 7 plasma AUC values for WAY-141624 from the customized SimPops after 100 mg BID dosing for 7 days compared to the maximum and minimum (dashed lines) Day 7 AUC observed in clinical study CK-LX1403.


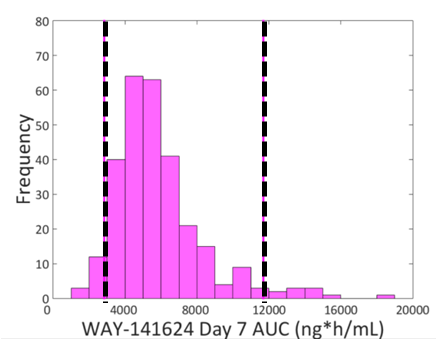


Figure A7. Distribution of Day 7 plasma AUC values for WAY-138758 from the customized SimPops after 100 mg BID dosing for 7 days compared to the maximum and minimum (dashed lines) Day 7 AUC observed in clinical study CK-LX1403.


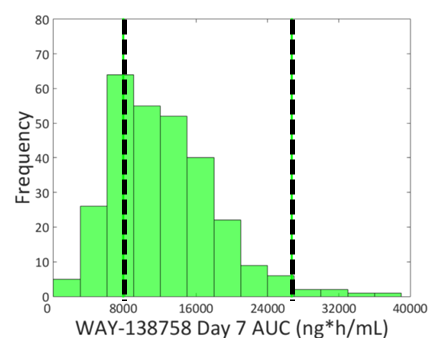


Table A1. Parameters used in the DILIsym Compound W PBPK sub-model for lixivaptan and its metabolites. The more complex Compound W scaffold was used because it allows for metabolite tracking. Note that values for the reactive metabolite parameters RM1 and RM2 are defined in this table. This does not mean that any of the metabolites for lixivaptan were represented as reactive metabolites; rather, the RM1 and RM2 scaffolds within DILIsym were used to represent stable metabolites of lixivaptan.

| DILIsym Parameter | Unit | Value |
| --- | --- | --- |
| Compound W absorption from gut | 1/hour | 451.5 |
| Compound W rate of elimination in feces | 1/hour | 0.54 |
| Compound W gut:blood | dimensionless | 2.86 |
| Compound W muscle:blood | dimensionless | 1.83 |
| Compound W other:blood | dimensionless | 2.7 |
| Compound W liver:blood | dimensionless | 5.34 |
| Compound W/Met A/Met B fraction unbound plasma | dimensionless | 2.82 x 10^-3^ |
| Compound W biliary clearance Vmax | μg/hour/kg^0.75^ | 7.91 x 10^7^ |
| Compound W renal clearance | mL/hour/kg^0.75^ | 3.8 x 10^4^ |
| Vmax (Compound W metabolite A) | nmol/hour/kg^0.75^ | 2.92 x 10^8^ |
| Vmax (Compound W metabolite B) | nmol/hour/kg^0.75^ | 3.5 x 10^7^ |
| Vmax (Compound W RM 1) | nmol/hour/kg^0.75^ | 1.61 x 10^7^ |
| Vmax (Compound W RM 2) | nmol/hour/kg^0.75^ | 3.1 x 10^7^ |
| Compound W Met A biliary clearance Vmax | μg/hour/kg^0.75^ | 1.22 x 10^9^ |
| Compound W Met A renal clearance Vmax | mL/hour/kg^0.75^ | 2.7 x 10^5^ |
| Compound W Met A volume of distribution by weight | mL/kg | 1504 |
| Compound W Met A liver:blood | dimensionless | 0.78 |
| Compound W Met B biliary clearance Vmax | μg/hour/kg^0.75^ | 2.9 x 10^7^ |
| Compound W Met B renal clearance Vmax | mL/hour/kg^0.75^ | 3132 |
| Compound W Met B volume of distribution by weight | mL/kg | 193.6 |
| Compound W Met B liver:blood | dimensionless | 0.024 |
| Compound W RM1 Adduct transport Vmax | nmol/mL/hour | 327 |
| Compound W RM2 Adduct transport Vmax | nmol/mL/hour | 2.75 x 10^4^ |
| Compound W RM1 Adduct half-life | hour | 6.6 |
| Compound W RM2 Adduct half-life | hour | 3.7 |

Table A2. PK parameters related to exposure of lixivaptan and its metabolites varied within the lixivaptan-specific custom SimPops, and their attendant ranges.

|  | **Compound W biliary Vmax** | **Compound W renal clearance** | **Comp-ound W k(ab)** | **Compound W k(out)** | **Met A biliary Vmax** | **Met A renal clearance** | **Metabolism scaling factor** | **Met B biliary Vmax** | **Met B renal clearance** | **RM1 transport Vmax** | **RM1 half life** |
| --- | --- | --- | --- | --- | --- | --- | --- | --- | --- | --- | --- |
| **Mean** | 7.91 x 10^7^ | 3.8 x 10^4^ | 451.5 | 0.54 | 1.22 x 10^9^ | 2.7 x 10^5^ | 1 | 2.9 x 10^7^ | 3132 | 327 | 6.6 |
| **SD/**  **mean** | 0.33 | 0.33 | 0.33 | 0.33 | 0.4 | 0.33 | 0.81 | 0.33 | 0.33 | 0.33 | 0.33 |
| **Max Z-score** | 2 | 2 | 2 | 2 | 2 | 2 | 6.7 | 2 | 2 | 2 | 2 |
| **Min Z-score** | -2 | -2 | -2 | -2 | -2 | -2 | -1 | -2 | -2 | -2 | -2 |

Table A3. SimPops documentation for Human_ROS_apop_mito_BA_v4A_1.

| Data used to Define Parameter Distributions (if applicable) | | |
| --- | --- | --- |
| Parameter Symbol in DILIsym | **Parameter Name in DILIsym** | **Data Source for Distribution** |
| ATP_decr_necrosis_Vmax | ATP decrement necrosis Vmax | Assumed standard deviation of ±20% and parameter range of 2.5 times the S.D. and validated with outcome data |
| Body_mass | Body Mass | Parameter range from NHANES III (human data) |
| GSH_pre_trans_Vmax | GSH precursor transport Vmax | Parameter range derived from (11) |
| GSHo | GSH basal level | Parameter range from (12,13) |
| HGF_prod_LSEC_Vmax | Maximum LSEC HGF production rate per liver LSEC | Assumed standard deviation of ±20% and parameter range of 2.5 times the S.D. and validated with outcome data |
| HGF_regen_Vmax | HGF mediated regeneration Vmax | Assumed standard deviation of ±20% and parameter range of 2.5 times the S.D. and validated with outcome data |
| RNS_ROS_ATP_inhib_Vmax | RNS/ROS ATP inhibition Vmax | Parameter range derived from (14) |
| RNS_ROS_cl_Vmax | Liver RNS/ROS baseline clearance Vmax | Assumed standard deviation of ±20% and parameter range of 2.5 times the S.D. and validated with outcome data |
| Basal_Stdzd_MitoETC_Flux | Basal value of mito ETC flux | Parameter range from healthy volunteer data (15) |
| Resp_Reserve_Scalar | Scaling coefficient representing reserve mitochondria function | Parameter range from healthy volunteer data (15) |
| CAS_apop_scale | Caspase-mediated apoptosis scaling constant | Parameter range derived from (16) |
| BA_uptake_Vmax | Bulk bile acid uptake Vmax | All transporters were assumed to have the same distribution as human BSEP reported in (17); similar expression ranges are also reported in (18); all uptake Vmax values are covariant |
| BA_baso_Vmax | Bulk bile acid basolateral transport Vmax | All transporters were assumed to have the same distribution as human BSEP reported in (17); similar expression ranges are also reported in (18); all basolateral Vmax values are covariant |
| BA_canal_Vmax | Bulk bile acid canalicular transport Vmax | All transporters were assumed to have the same distribution as human BSEP reported in (17); similar expression ranges are also reported in (18); all canalicular Vmax values are covariant |
| LCA_uptake_Vmax | LCA uptake Vmax | All transporters were assumed to have the same distribution as human BSEP reported in (17); similar expression ranges are also reported in (18); all uptake Vmax values are covariant |
| LCA_baso_Vmax | LCA basolateral transport Vmax | All transporters were assumed to have the same distribution as human BSEP reported in (17); similar expression ranges are also reported in (18); all basolateral Vmax values are covariant |
| LCA_canal_Vmax | LCA canalicular transport Vmax | All transporters were assumed to have the same distribution as human BSEP reported in (17); similar expression ranges are also reported in (18); all canalicular Vmax values are covariant |
| LCAamide_uptake_Vmax | LCA-amide uptake Vmax | All transporters were assumed to have the same distribution as human BSEP reported in (17); similar expression ranges are also reported in (18); all uptake Vmax values are covariant |
| LCAamide_baso_Vmax | LCA-amide basolateral transport Vmax | All transporters were assumed to have the same distribution as human BSEP reported in (17); similar expression ranges are also reported in (18); all basolateral Vmax values are covariant |
| LCAamide_canal_Vmax | LCA-amide canalicular transport Vmax | All transporters were assumed to have the same distribution as human BSEP reported in (17); similar expression ranges are also reported in (18); all canalicular Vmax values are covariant |
| LCAsulfate_uptake_Vmax | LCA-sulfate uptake Vmax | All transporters were assumed to have the same distribution as human BSEP reported in (17); similar expression ranges are also reported in (18); all uptake Vmax values are covariant |
| LCAsulfate_baso_Vmax | LCA-sulfate basolateral transport Vmax | All transporters were assumed to have the same distribution as human BSEP reported in (17); similar expression ranges are also reported in (18); all basolateral Vmax values are covariant |
| LCAsulfate_canal_Vmax | LCA-sulfate canalicular transport Vmax | All transporters were assumed to have the same distribution as human BSEP reported in (17); similar expression ranges are also reported in (18); all canalicular Vmax values are covariant |
| CDCA_uptake_Vmax | CDCA uptake Vmax | All transporters were assumed to have the same distribution as human BSEP reported in (17); similar expression ranges are also reported in (18); all uptake Vmax values are covariant |
| CDCA_baso_Vmax | CDCA basolateral transport Vmax | All transporters were assumed to have the same distribution as human BSEP reported in (17); similar expression ranges are also reported in (18); all basolateral Vmax values are covariant |
| CDCA_canal_Vmax | CDCA canalicular transport Vmax | All transporters were assumed to have the same distribution as human BSEP reported in (17); similar expression ranges are also reported in (18); all canalicular Vmax values are covariant |
| CDCAamide_uptake_Vmax | CDCA-amide uptake Vmax | All transporters were assumed to have the same distribution as human BSEP reported in (17); similar expression ranges are also reported in (18); all uptake Vmax values are covariant |
| CDCAamide_baso_Vmax | CDCA-amide basolateral transport Vmax | All transporters were assumed to have the same distribution as human BSEP reported in (17); similar expression ranges are also reported in (18); all basolateral Vmax values are covariant |
| CDCAamide_canal_Vmax | CDCA-amide canalicular transport Vmax | All transporters were assumed to have the same distribution as human BSEP reported in (17); similar expression ranges are also reported in (18); all canalicular Vmax values are covariant |
| CDCA_amidation_Vmax | CDCA amidation Vmax | Given same range as transporters due to lack of quantitative data |
| LCA_synthesis_Vmax | LCA synthesis Vmax | Assumed parameter range of ±2 orders of magnitude with ±50% standard deviation and validated with outcome data |
| LCAamide_sulfation_Vmax | LCA-amide sulfation Vmax | Given same range as transporters due to lack of quantitative data |
| canal_reg_scale | Canalicular transporter regulation exponent | Assumed parameter range of 0-8 with ±50% standard deviation and validated with outcome data |
| uptake_reg_scale | Uptake transporter regulation exponent | Assumed parameter range of 0-8 with ±50% standard deviation and validated with outcome data |

Table A4. Ratio of simulated vs. observed (Clinical Study 0892A1-109-US) amount of lixivaptan and its metabolites cleared renally and fecally after a single 100 mg dose.


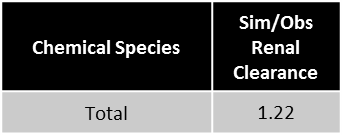

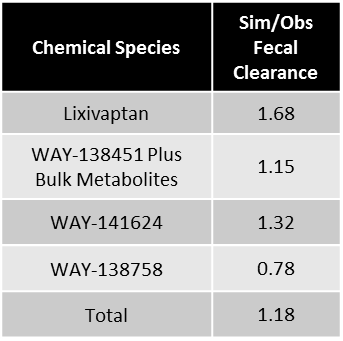


Table A5. Ratio of simulated vs. observed (Clinical Study CK-LX1403) pharmacokinetic parameters for lixivaptan following a 400 mg BID dosing regimen for 7 days. AUC was measured on day 7.

| **Chemical Species** | **Sim/Obs** | |
| --- | --- | --- |
|  | **Day 7 C_max_** | **Day 7 AUC** |
| Lixivaptan | 1.77 | 0.90 |
