# Supplementary material for: Comparison of the Hepatotoxic Potential of Two Treatments for Autosomal-Dominant Polycystic Kidney DiseaseUsing Quantitative Systems Toxicology Modeling
Source: Pharm Res. 2020 Jan 6;37(2):24. doi: 10.1007/s11095-019-2726-0 (PMC6944674; doi:10.1007/s11095-019-2726-0)
Supplement: Supplementary file 2 — (DOCX 1221 kb) [file 11095_2019_2726_MOESM2_ESM.docx]

# Supplement B: In Vitro Data Collection and Toxicity Parameter Determination

## Assay Description

### Bile acid transporter inhibition by lixivaptan, its metabolites, and tolvaptan

The functional impact of lixivaptan, its three metabolites and tolvaptan on the human ABC (efflux) transporters BSEP and MRP3 and MRP4 was assessed for this project using *in vitro* vesicular transport inhibition assays. The inhibition of the human SLC (uptake) transporter NTCP was assessed using CHO cells stably expressing human NTCP. The concentration ranges used for the five test articles was determined based on the solubility of each compound in the assay solution. The concentration ranges used for each assay are listed in Table B1. All transporter inhibition assays were conducted in triplicate by Solvo Biotechnology (Szeged, Hungary).

For the simulation of bile acid transporter inhibition by lixivaptan in DILIsym, parameter values were selected for BSEP canalicular transport, basolateral transport (encompassing both MRP3 and MRP4), and NTCP transport. The parameter values for each include the inhibition constant, defined as the IC_50_ or K_i_ value, and the inhibition type, defined as competitive, non-competitive, or mixed inhibition. For a given inhibition constant, the inhibition type has been shown to influence bile acid accumulation (4,5).

### Mitochondria toxicity due to lixivaptan, its metabolites, and tolvaptan

To assess the potential effect of the five test articles on mitochondrial function, cellular respiration assays using a Seahorse XFe96 Flux Analyzer were conducted by Cyprotex (Macclesfield, UK) and overseen by the authors. HepG2 cells were incubated with each test article for 1 or 24 hours and tested for an effect on cellular respiration by measuring the oxygen consumption rate (OCR). The concentration ranges used for each test article are listed in Table B1. Each study was conducted in triplicate. Importantly, the 1 hour culture was conducted in the absence of serum (i.e. no serum protein binding) as is standard for the Seahorse Analyzer assay conditions. Conversely, for the 24 hour assessment, given that prolonged HepG2 incubations in the absence of serum do not typically yield robust cell cultures, serum was present for the first 23 hours up to 1 hour before the assay, at which point cell culture media was exchanged for the standard assay media without serum.

The ETC inhibition parameter values for lixivaptan, WAY-138451, and tolvaptan were determined by reproducing the cellular respiration data within MITOsym®, a platform model of *in vitro* mitochondrial toxicity (10). The parameters calculated in this manner were then translated to DILIsym parameters using a conversion factor based on the relative activity of rotenone in the *in vitro* vs. *in vivo* environment (7).

The concentration of test compound at the site of action (i.e. hepatocytes) has been identified as highly influential in the selection of parameter values (7). To assess intracellular concentrations of the five test articles, identical HepG2 cultures were established in parallel and cell lysate concentrations of the test articles were measured by LC/MS/MS analysis. Intracellular concentrations of the test articles were calculated by correcting for cell lysate volume (100 µL) and the volume of HepG2 cells in each well, which was calculated by multiplying cell count per well by cellular volume per cell (2.85 pl/cell). One independent study in duplicate was conducted to assess intracellular concentrations of the test articles for Seahorse studies.

### Oxidative stress due to lixivaptan and its metabolites

To assess the potential for lixivaptan and its metabolites to induce oxidative stress, assays to measure formation of reactive oxygen species (ROS) were conducted by Cyprotex and overseen by the authors. HepG2 cells were cultured in triplicate in the presence of each test article for 1 or 24 hours; the range of concentrations used for each test article is listed in Table B1. ROS formation was assayed by DHE fluorescence. Two independent studies in triplicate were conducted on HepG2 cells.

Parameter values for lixivaptan- and WAY-138451-mediated induction of oxidative stress were identified by simulating the experimental data in DILIsym using a DILIsym dosing scheme meant to represent *in vitro* conditions (constant liver exposure).

To assess the intracellular concentration of lixivaptan and its metabolites, an identical HepG2 culture was established in parallel and cell lysate concentrations of each test article were measured by LC/MS/MS analysis. Intracellular concentrations of the compounds were calculated by correcting for cell lysate volume (100 µL) and the volume of HepG2 cells in each well, which was calculated by multiplying cell count per well by cellular volume per cell (2.85 pl/cell for HepG2). One independent study in triplicate was conducted to assess intracellular concentrations of the test articles for the ROS (high-content imaging) studies.

# Assay Results

### Bile acid transporter inhibition by lixivaptan and its metabolites

In vesicular transport inhibition assays, lixivaptan and its metabolites each inhibited BSEP-mediated probe substrate accumulation (Figure B1) and MRP4-mediated probe substrate accumulation (Figure B2) dose-dependently. The three metabolites, but not the parent, inhibited MRP3-mediated probe substrate accumulation (Figure B3). Inhibition of NTCP-mediated probe substrate accumulation (Figure B4) was observed for lixivaptan, WAY-141624, and WAY-138758. IC_50_ values were calculated for each compound for each transporter they inhibited; these values are shown in Table 11. In cases where 50% inhibition was not observed, IC_50_ values were calculated from available data by curve-fitting and extrapolation. For comparison, the mean C_max_ of lixivaptan after 7 days of dosing with 100 mg BID doses was 2 μM in clinical study CK-LX1403 (data not shown).

### Bile acid transporter inhibition by tolvaptan

Vesicular transport inhibition assays showed that tolvaptan inhibited BSEP (Figure B5), MRP3 (Figure B6), and NTCP (Figure B7), as previously reported (7). In this study, the calculated IC_50_ value for BSEP inhibition by tolvaptan was 16.5 μM; a somewhat higher measured K_i_ value for tolvaptan was used in DILIsym simulations previously (31.6 μM; see reference (7)).

### Mitochondrial toxicity due to lixivaptan and its metabolites

In the mitochondrial respiration assay, a 24 hour incubation with lixivaptan or WAY-138451 was found to reduce the HepG2 oxygen consumption rate (OCR) in a dose-dependent manner (Figure B8). This suggests that lixivaptan and WAY-138451 both act as mitochondrial ETC inhibitors. The effect of WAY-138451 was more pronounced than that of lixivaptan, whereas the other two metabolites, WAY-141624 and WAY-138758, did not appear to have a substantial effect on mitochondrial function. Intracellular concentrations of lixivaptan (Table B2) and WAY-138451 (Table B3) in identically cultured HepG2 cells were measured by LC/MS/MS analysis and were used to define the quantitative relationship between intracellular exposure and OCR responses.

### Mitochondrial toxicity due to tolvaptan

In the mitochondrial respiration assay, a 24-hour incubation with tolvaptan was found to reduce the HepG2 OCR in a dose-dependent manner (Figure B9). The inhibition observed in this assay is qualitatively similar to that observed previously for tolvaptan (7), once again validating the mitochondrial toxicity assay conducted in this study. These data were used in conjunction with the intracellular concentrations of tolvaptan in identically cultured HepG2 cells measured by LC/MS/MS analysis (Table B4) in order to calculate the DILIsym ETC inhibition toxicity parameter for tolvaptan (discussed below).

### Oxidative stress due to lixivaptan and its metabolites

In the oxidative stress assay, there was evidence for induction of oxidative stress in HepG2 cells following 24 hour incubation with lixivaptan or WAY-138451; neither WAY-141624 nor WAY-138758 induced oxidative stress (Figure B10). The 24 hour incubation data was utilized to identify the corresponding DILIsym parameter in conjunction with the intracellular concentrations of lixivaptan and WAY-138451 measured by LC/MS/MS analysis in identically cultured HepG2 cells (Tables B2 and B3).

# Toxicity Parameter Determination

### Bile acid transporter inhibition by lixivaptan and its metabolites

The estimated IC_50_ values for lixivaptan and its metabolites listed in the main manuscript Table 2 were used as the K_i_ values in DILIsym; while IC_50_ and K_i_ values can differ, this approximation is reasonable when the assay substrate concentration is well below the K_m_ for the transporter, as is the case for these studies. Because K_i_ studies were not performed for the bile acid transporters, mixed inhibition with alpha of 5 was assumed for all transporters. This assumption was validated by sensitivity analyses simulations conducted to determine if the mode of bile acid transporter inhibition was an important factor; the results of these simulations are not shown here.

### Mitochondrial toxicity parameters for tolvaptan, lixivaptan, and WAY-138451

To define the DILIsym parameter values for tolvaptan-, lixivaptan-, and WAY-138451-mediated mitochondrial toxicity, the 24 hour *in vitro* data were simulated within MITOsym (Figure B11) and subsequently translated to DILIsym values as described in the Methods section. The intracellular concentration of tolvaptan, lixivaptan, and WAY-138451 at each dose was measured by LC/MS/MS analysis and was employed in parameterization. Reproduction of each compound’s inhibition of mitochondrial respiration (as measured by oxygen consumption rate) in DILIsym defined them all as potential electron transport chain (ETC) inhibitors. The calculated DILIsym parameter of 729 µM for tolvaptan for this work is 35% smaller than the parameter used in previously published research, which was 1.09 mM (9). The difference is likely due to the fact that intracellular concentrations are available for this work while they were not measured for prior published work (9). This more potent ETC inhibition coefficient would likely have led to an increased incidence of simulated ALT elevations for tolvaptan had it been used in the published work. Calculated parameters for lixivaptan (represented by ETC inhibition 1) and WAY-138451 (represented by ETC inhibition 2) are listed in Table 2; they are both predicted to be mildly more potent inhibitors than tolvaptan. WAY-141624 and WAY-138758 had no effect on mitochondrial respiration (Supplement B).

### Oxidative stress due to lixivaptan and WAY-138451

To define the DILIsym parameter values for lixivaptan- and WAY-138451-mediated oxidative stress, the 24 hour *in vitro* data were simulated within DILIsym (Figure B12). The intracellular concentration of each compound was measured by LC/MS/MS analysis. For lixivaptan, the measured intracellular concentration at each dose was used to parameterize the model. For WAY-138451, however, the LC/MS/MS data was inconsistent at low doses due to lower limit of detection issues. Thus, the mean measured cell-to-nominal-media ratio (0.672) was used to estimate intracellular concentrations at each dose. Reproduction of lixivaptan- and WAY-138451-mediated induction of oxidative stress defined the relationship between liver compound concentration and ROS formation with the reactive nitrogen/oxygen species (RNS/ROS) production rate constants listed in Table 2. WAY-141624 and WAY-138758 had no effect on cellular oxidative stress.

Figure B1. Inhibition of BSEP-mediated taurocholate transport into HEK-293 cell membrane vesicles observed for a) lixivaptan; b) WAY-138451; c) WAY-141624; and d) WAY-138758. See Appendix A for more details.


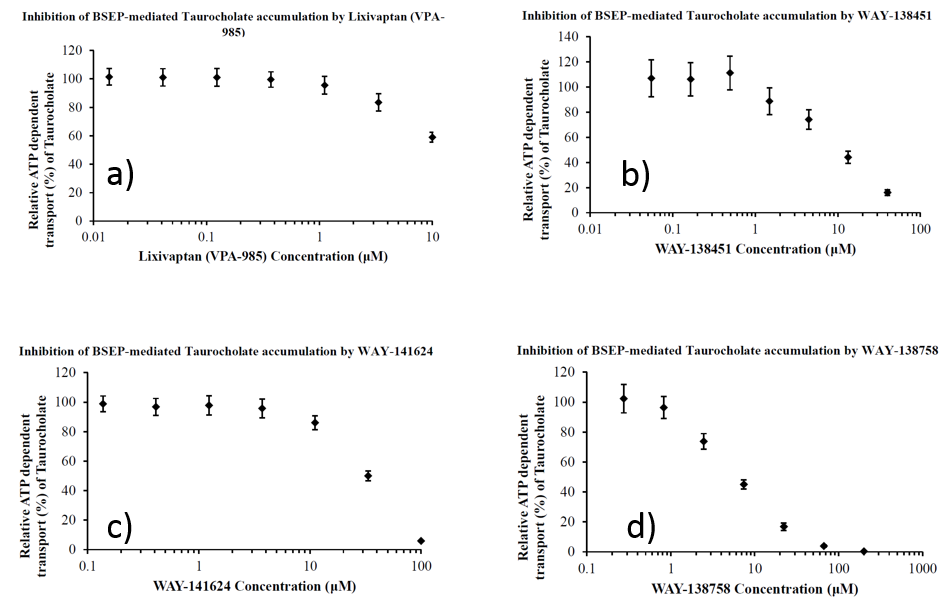


Figure B2. Inhibition of MRP4-mediated E217βG transport into HEK-293 cell membrane vesicles observed for a) lixivaptan; b) WAY-138451; c) WAY-141624; and d) WAY-138758. See Appendix A for more details.


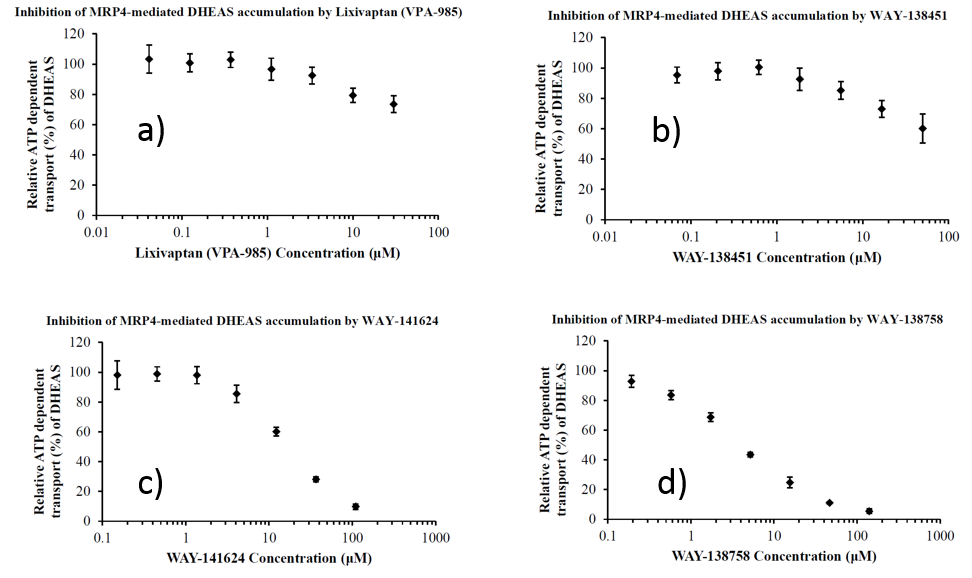


Figure B3. Inhibition of MRP3-mediated E217βG transport into HEK-293 cell membrane vesicles observed for a) lixivaptan; b) WAY-138451; c) WAY-141624; and d) WAY-138758. See Appendix A for more details.


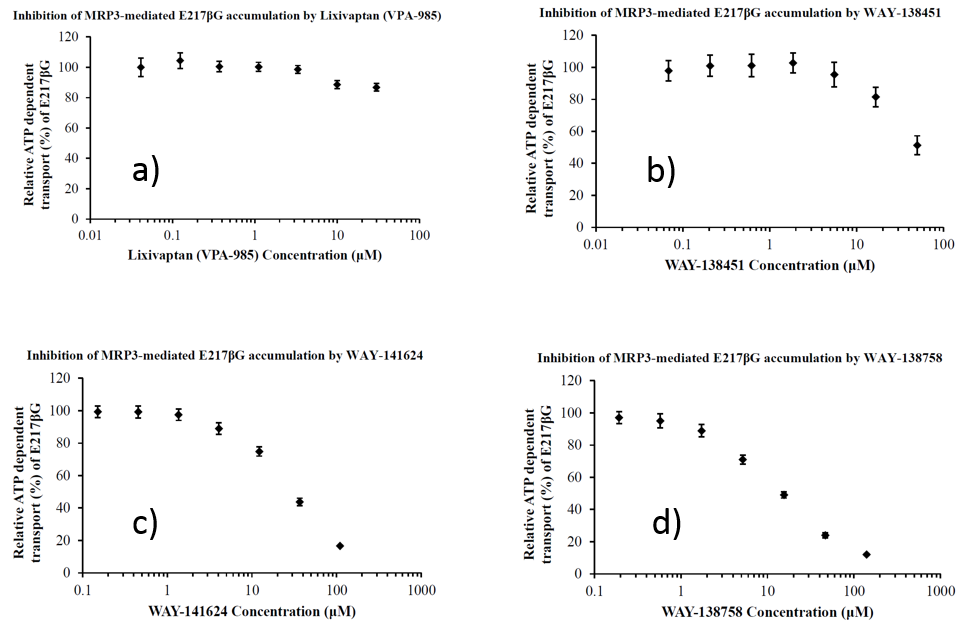


Figure B4. Inhibition of NTCP-mediated taurocholate transport into CHO cells observed for a) lixivaptan; b) WAY-138451; c) WAY-141624; and d) WAY-138758. See Appendix A for more details.


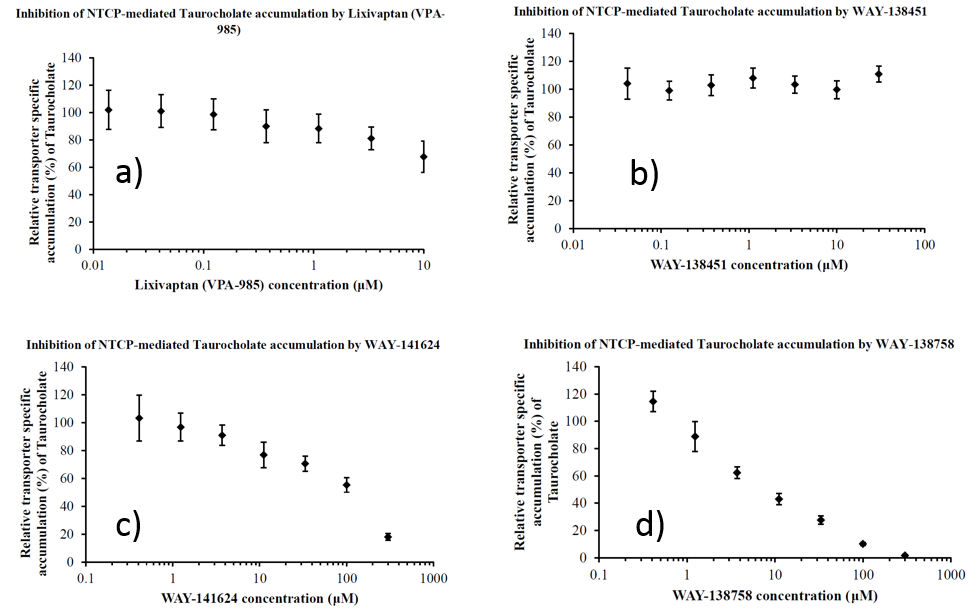


Figure B5. Inhibition of BSEP-mediated taurocholate transport into HEK-293 cell membrane vesicles observed for tolvaptan. See Appendix A for more details.


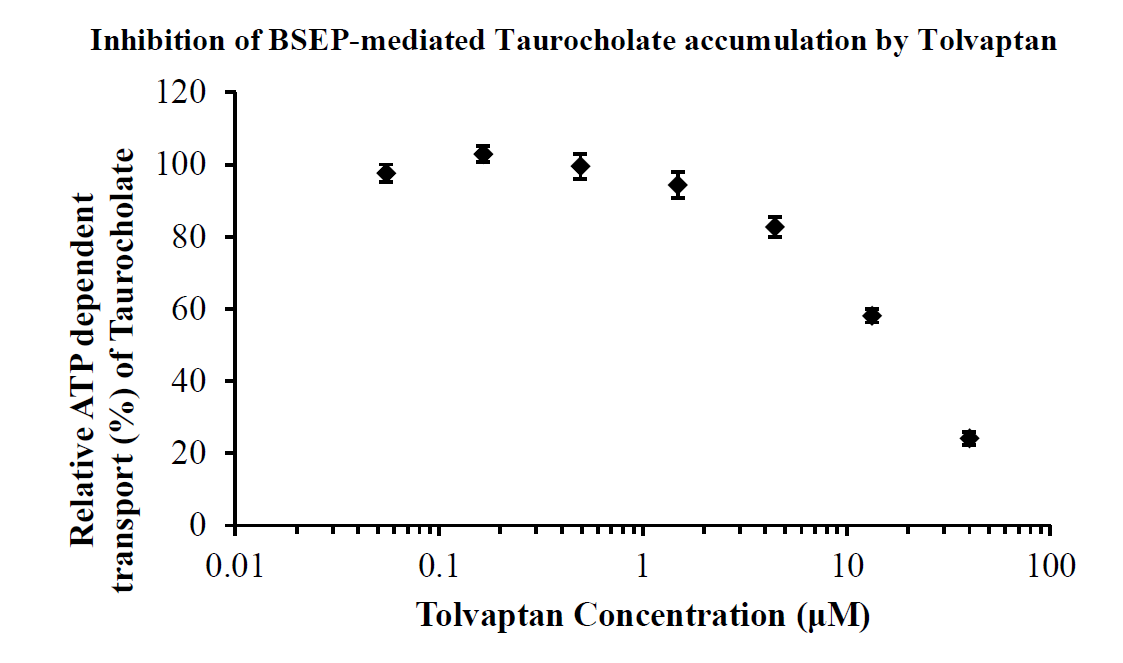


Figure B6. Inhibition of MRP3-mediated E217βG transport into HEK-293 cell membrane vesicles observed for tolvaptan. See Appendix A for more details.


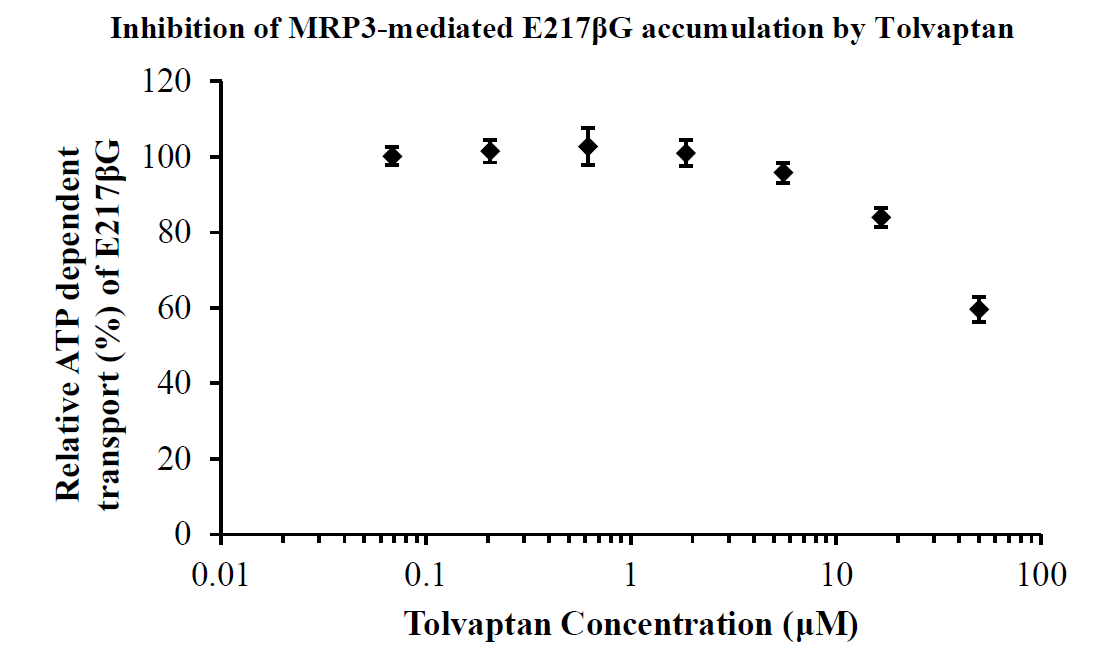


Figure B7. Inhibition of NTCP-mediated taurocholate transport into CHO cells observed for tolvaptan. See Appendix A for more details.


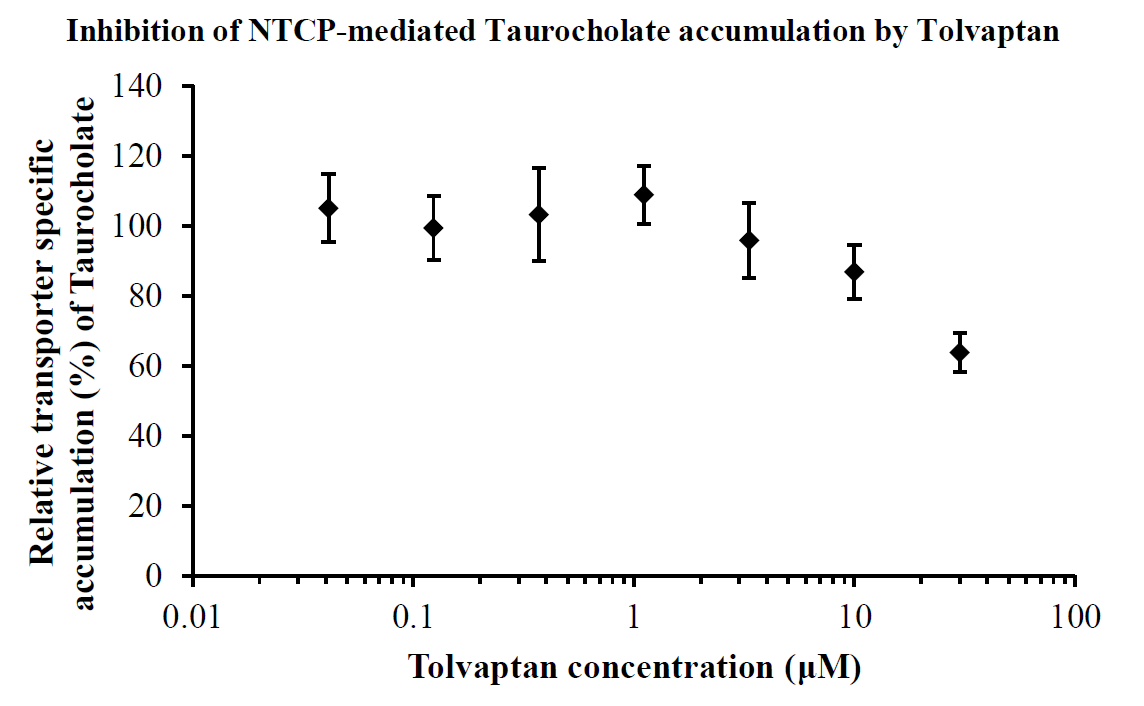


Figure B8. Dose-dependent effect of lixivaptan and its metabolites on the oxygen consumption rate (OCR) of HepG2 cells after a 24-hour incubation.


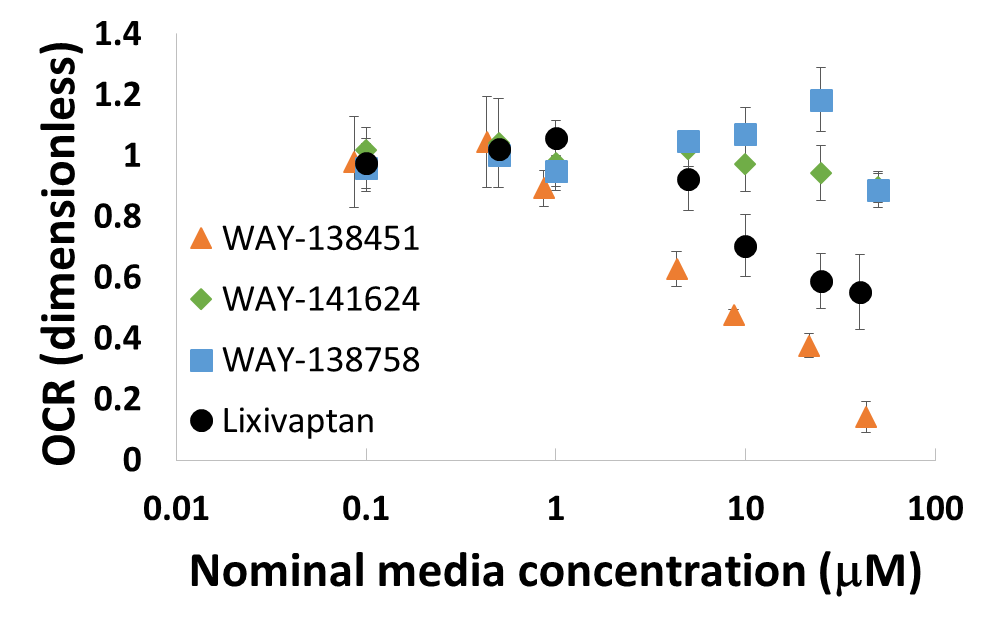


Figure B9. Dose-dependent effect of tolvaptan on the oxygen consumption rate (OCR) of HepG2 cells after a 24-hour incubation.


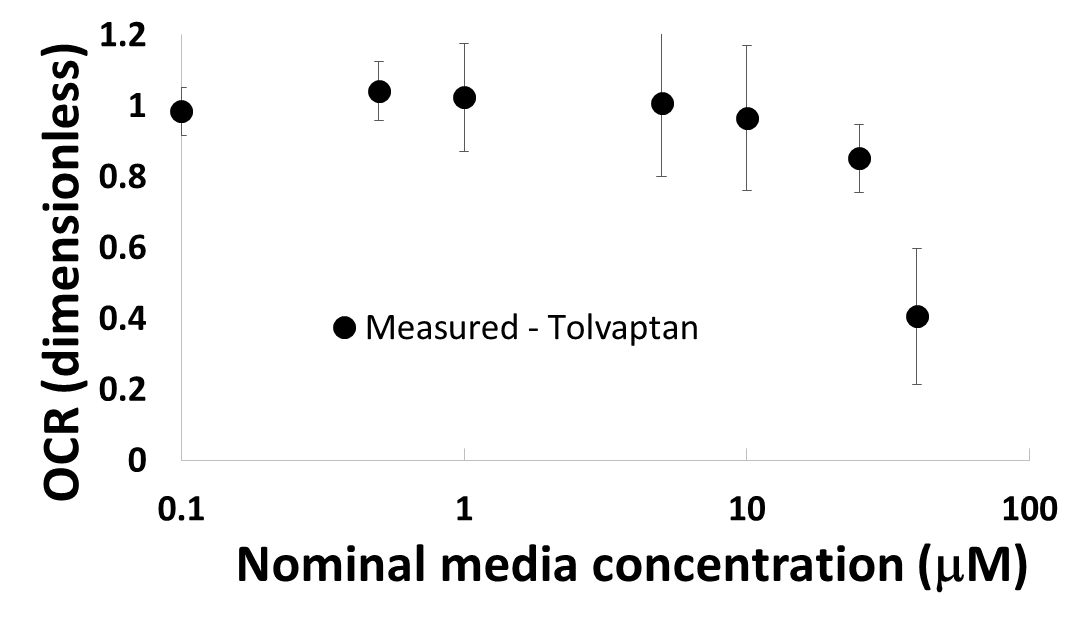


Figure B10. Generation of oxidative stress in HepG2 cells by lixivaptan and its metabolites after a 24-hour incubation.


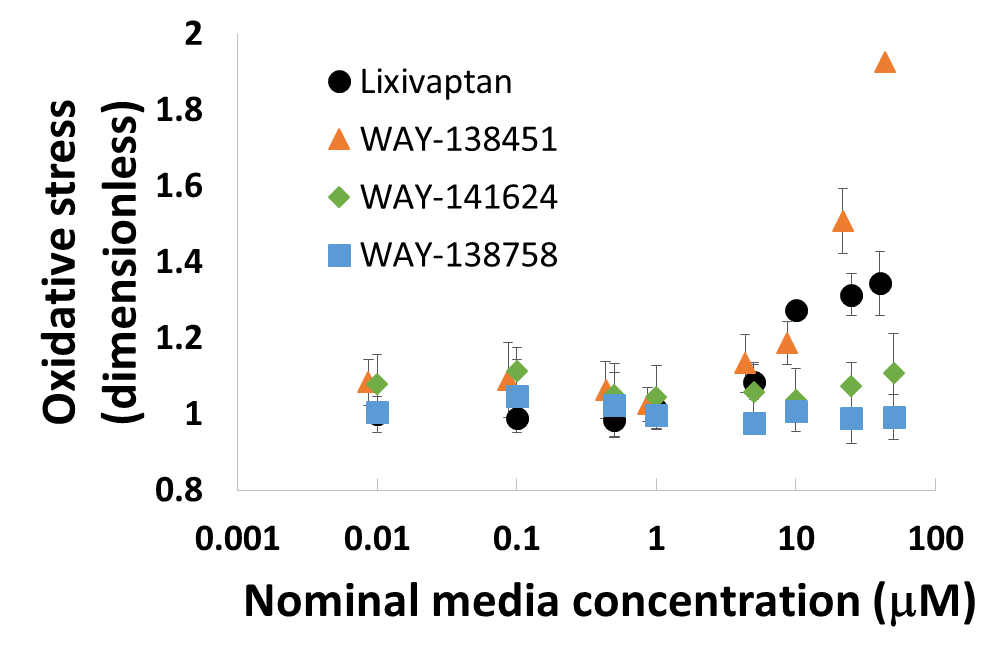


Figure B11. MITOsym simulations used to calculate ETC inhibition parameters for a) lixivaptan; b) its WAY-138451 metabolite; and c) tolvaptan, compared with measured 24 hr HepG2 data. The OCR data are reported as fraction of the control experiment (see Supplement B).


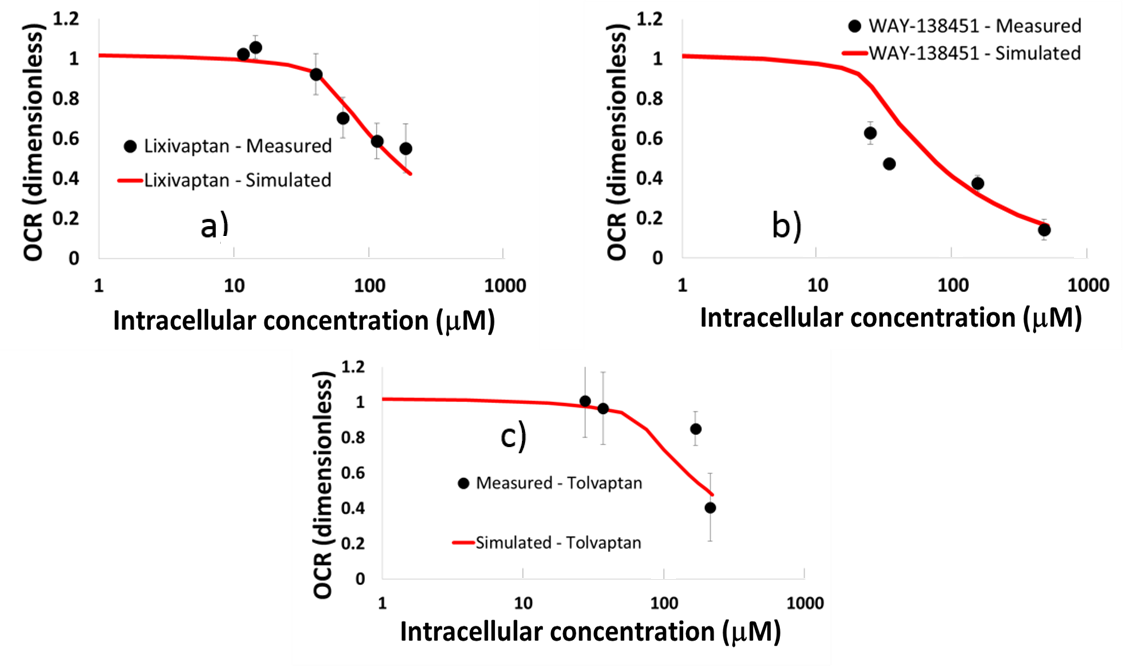


Figure B12. DILIsym simulations used to calculate ROS generation parameters for a) lixivaptan; and b) its WAY-138451 metabolite, compared with measured 24 hr HepG2 data. For WAY-138451, the gray dots denote intracellular concentrations calculated by extrapolating the average measured ratio between nominal media and intracellular concentration for the two highest WAY-138451 concentrations to all concentrations for which ROS was measured.


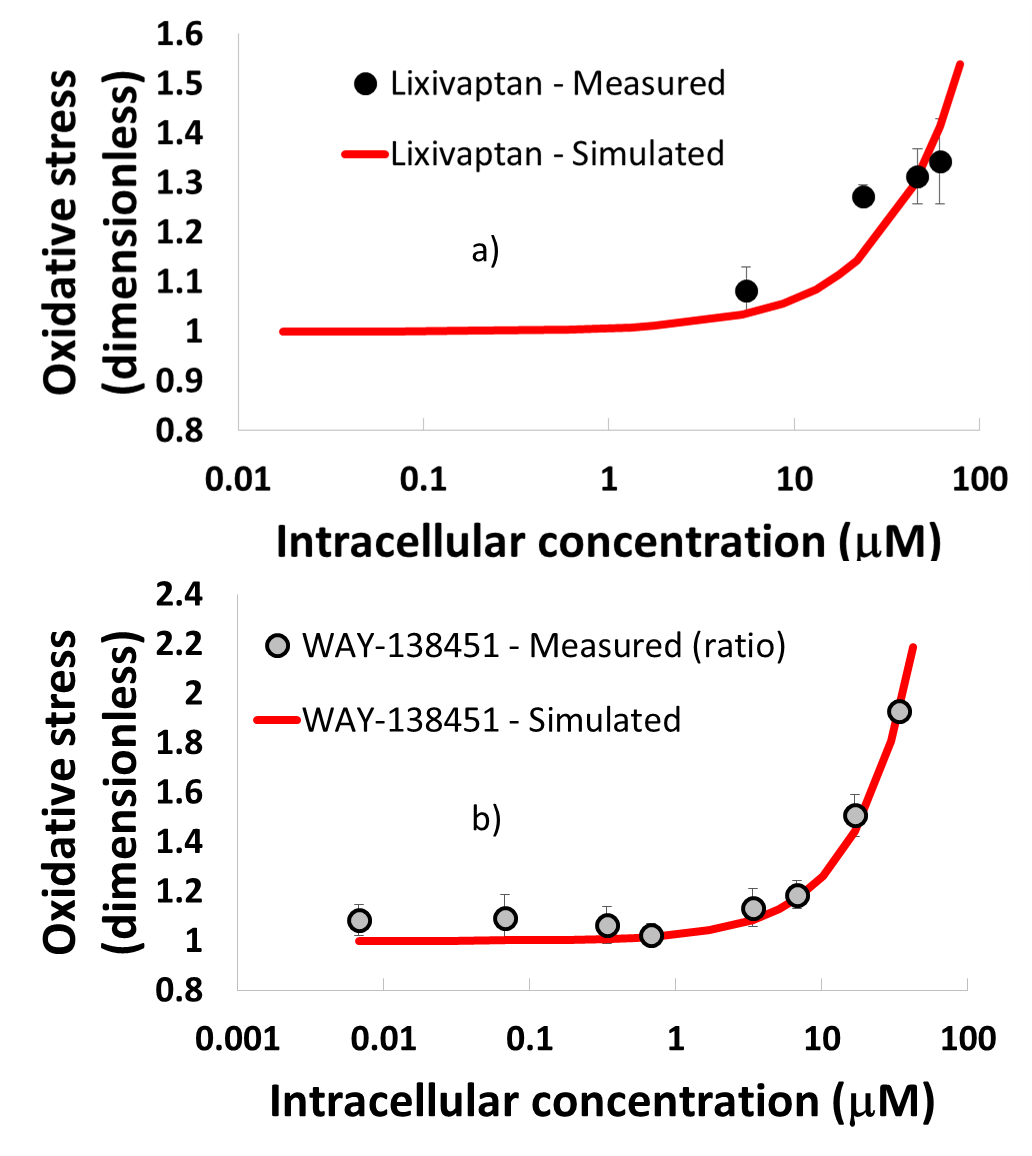


Table B1. Concentration ranges used for each *in vitro* assay for lixivaptan, its major metabolites, and tolvaptan. All concentrations are expressed in μM.

|  | Lixivaptan | WAY-138451 | WAY-141624 | WAY-138758 | Tolvaptan |
| --- | --- | --- | --- | --- | --- |
| ROS assay | 0.1-40 | 0.0865-43.25 | 0.1-50 | 0.1-50 | 0.1-40 |
| Seahorse assay | 0.1-40 | 0.0865-43.25 | 0.1-50 | 0.1-50 | 0.1-40 |
| BSEP vesicle assay | 0.01-10* | 0.05-40 | 0.14-100 | 0.27-200 | 0.05-40 |
| MRP3/4 vesicle assay | 0.01-30 | 0.07-50 | 0.15-110 | 0.19-140 | 0.07-50 |
| NTCP CHO cell assay | 0.01-10* | 0.04-30 | 0.41-300 | 0.41-300 | 0.04-30 |

* Upper range limited by test article solubility in assay buffer

Table B2. Intracellular concentrations of lixivaptan in HepG2 cells in the cellular respiration assay and high-content screening assay used to determine oxidative stress levels.

| Incubation Medium | Dose (µM) | C_Extracellular Fraction_ (µM) | C_Intracellular Fraction_ (µM) | Cell count (cells/ well) | V_hepatocytes_ (µl/well)) | C_Hepatocyte_ (µM) | Kp |
| --- | --- | --- | --- | --- | --- | --- | --- |
| Seahorse* | 0.1 | NA | 0 | 17370 | 0.0495 | 0 | NC |
|  | 0.5 | NA | 0.00582 | 17567.5 | 0.0501 | 11.61 | 23.23 |
|  | 1 | NA | 0.00738 | 18071 | 0.0515 | 14.34 | 14.34 |
|  | 5 | NA | 0.0193 | 16891.5 | 0.0481 | 40.10 | 8.020 |
|  | 10 | NA | 0.0307 | 16953.5 | 0.0483 | 63.51 | 6.351 |
|  | 25 | NA | 0.0525 | 16310 | 0.0465 | 112.99 | 4.520 |
|  | 40 | NA | 0.0823 | 15766.5 | 0.0449 | 184.62 | 4.62 |
| High content screening | 0.01 | 0.000 ± 0.000 | 0.000 ± 0.000 | 9434 ± 699 | 0.027 ± 0.002 | 0.000 ± 0.000 | NC |
|  | 0.1 | 0.001 ± 0.001 | 0.000 ± 0.000 | 10382 ± 1414 | 0.030 ± 0.004 | 0.000 ± 0.000 | NC |
|  | 0.5 | 0.020 ± 0.001 | 0.000 ± 0.000 | 9794 ± 948 | 0.028 ± 0.003 | 0.000 ± 0.000 | NC |
|  | 1 | 0.047 ± 0.001 | 0.000 ± 0.000 | 9457 ± 594 | 0.027 ± 0.002 | 0.000 ± 0.000 | NC |
|  | 5 | 0.345 ± 0.011 | 0.0013 ± 0.0016 | 8932 ± 929 | 0.025 ± 0.003 | 6.02 ± 6.30 | 17.56 ± 18.44 |
|  | 10 | 0.736 ± 0.055 | 0.0054 ± 0.0032 | 8011 ± 250 | 0.023 ± 0.001 | 28.44 ± 5.12 | 39.74 ± 6.49 |
|  | 25 | 2.751 ± 0.143 | 0.0093 ± 0.0055 | 7001 ± 733 | 0.020 ± 0.002 | 55.57 ± 15.10 | 19.91 ± 4.54 |
|  | 40 | 5.773 ± 0.508 | 0.0114 ± 0.0058 | 6433 ± 587 | 0.018 ± 0.002 | 71.75 ± 11.05 | 12.94 ± 2.68 |

*Standard deviation (SD) not calculated for seahorse samples because experiments performed in duplicate

Table B3. Intracellular concentrations of WAY-138451 in HepG2 cells in the cellular respiration assay and high-content screening assay used to determine oxidative stress levels.

| Incubation Medium | Dose (µM) | C_Extracellular Fraction_ (µM) | C_Intracellular Fraction_ (µM) | Cell count (cells/ well) | V_hepatocytes_ (µl/well)) | C_Hepatocyte_ (µM) | Kp |
| --- | --- | --- | --- | --- | --- | --- | --- |
| Seahorse* | 0.1 | NA | 0 | 18811.5 | 0.0536 | 0 | NC |
|  | 0.5 | NA | 0 | 15619.5 | 0.0445 | 0 | NC |
|  | 1 | NA | 0 | 16343 | 0.0466 | 0 | NC |
|  | 5 | NA | 0.0114 | 16414.5 | 0.0468 | 24.46 | 4.89 |
|  | 10 | NA | 0.0158 | 16508.5 | 0.0470 | 33.57 | 3.36 |
|  | 25 | NA | 0.0628 | 14485.5 | 0.0413 | 151.84 | 6.07 |
|  | 50 | NA | 0.181 | 13530 | 0.0386 | 469.74 | 9.39 |
| High content Screening | 0.01 | 0.0000 ± 0.0000 | 0.000 ± 0.000 | 6213 ± 219 | 0.0177 ± 0.0006 | 0.000 ± 0.000 | NC |
|  | 0.1 | 0.0231 ± 0.0029 | 0.000 ± 0.000 | 6454 ± 133 | 0.0184 ± 0.0004 | 0.000 ± 0.000 | NC |
|  | 0.5 | 0.1630 ± 0.0087 | 0.000 ± 0.000 | 6400 ± 346 | 0.0182 ± 0.0010 | 0.000 ± 0.000 | NC |
|  | 1 | 0.3332 ± 0.0223 | 0.000 ± 0.000 | 6341 ± 270 | 0.0181 ± 0.0008 | 0.000 ± 0.000 | NC |
|  | 5 | 1.7698 ± 0.1441 | 0.000 ± 0.000 | 6062 ± 112 | 0.0173 ± 0.0003 | 0.000 ± 0.000 | NC |
|  | 10 | 3.0632 ± 0.0323 | 0.000 ± 0.000 | 5674 ± 141 | 0.0162 ± 0.0004 | 0.000 ± 0.000 | NC |
|  | 25 | 6.7587 ± 0.2036 | 0.0005 ± 0.0004 | 4295 ± 150 | 0.0122 ± 0.0004 | 3.77 ± 3.27 | 0.55 ± 0.48 |
|  | 50 | 12.1685 ± 1.0148 | 0.0033 ± 0.0003 | 2406 ± 222 | 0.0069 ± 0.0006 | 48.52 ± 7.34 | 4.04 ± 0.97 |

*Standard deviation (SD) not calculated for seahorse samples because experiments performed in duplicate

Table B4. Intracellular concentrations of tolvaptan in HepG2 cells in the cellular respiration assay.

| Incubation Medium | Dose (µM) | C_Extracellular Fraction_ (µM) | C_Intracellular Fraction_ (µM) | Cell count (cells/ well) | V_hepatocytes_ (µl/well)) | C_Hepatocyte_ (µM) | Kp |
| --- | --- | --- | --- | --- | --- | --- | --- |
| Seahorse* | 0.1 | NA | 0 | 18168.5 | 0.0518 | 0 | NC |
|  | 0.5 | NA | 0 | 17070 | 0.0486 | 0 | NC |
|  | 1 | NA | 0 | 17095 | 0.0487 | 0 | NC |
|  | 5 | NA | 0.0156 | 17036.5 | 0.0485 | 32.20 | 6.44 |
|  | 10 | NA | 0.0162 | 16585 | 0.0473 | 34.40 | 3.44 |
|  | 25 | NA | 0.0629 | 14743.5 | 0.0420 | 149.2 | 5.97 |
|  | 40 | NA | 0.0937 | 14390 | 0.0410 | 227.9 | 5.70 |

*Standard deviation (SD) not calculated for seahorse samples because experiments performed in duplicate
